# Mechanistic and quantitative insight into cell surface targeted molecular imaging agent design

**DOI:** 10.1038/srep25424

**Published:** 2016-05-05

**Authors:** Liang Zhang, Sumit Bhatnagar, Emily Deschenes, Greg M. Thurber

**Affiliations:** 1 Department of Chemical Engineering, University of Michigan, Ann Arbor, MI 48109, US; 2 Department of Biomedical Engineering, University of Michigan, Ann Arbor, MI 48109, US.

## Abstract

Molecular imaging agent design involves simultaneously optimizing multiple probe properties. While several desired characteristics are straightforward, including high affinity and low non-specific background signal, in practice there are quantitative trade-offs between these properties. These include plasma clearance, where fast clearance lowers background signal but can reduce target uptake, and binding, where high affinity compounds sometimes suffer from lower stability or increased non-specific interactions. Further complicating probe development, many of the optimal parameters vary depending on both target tissue and imaging agent properties, making empirical approaches or previous experience difficult to translate. Here, we focus on low molecular weight compounds targeting extracellular receptors, which have some of the highest contrast values for imaging agents. We use a mechanistic approach to provide a quantitative framework for weighing trade-offs between molecules. Our results show that specific target uptake is well-described by quantitative simulations for a variety of targeting agents, whereas non-specific background signal is more difficult to predict. Two *in vitro* experimental methods for estimating background signal *in vivo* are compared – non-specific cellular uptake and plasma protein binding. Together, these data provide a quantitative method to guide probe design and focus animal work for more cost-effective and time-efficient development of molecular imaging agents.

Targeting cell surface receptors provides a robust method for identifying abnormal tissue, monitoring disease progression, and quantifying therapeutic response^1^. Typically, a detectable label (e.g. radioactive isotope, fluorescent dye, MRI, CT, or ultrasound contrast agent) is chemically conjugated to a targeting ligand to create the imaging probe. These agents are then administered to a patient or animal to target tissues that express antigens specific to the targeting ligand. Monitoring the imaging compound *in vivo* provides valuable insight into diverse biological processes such as cell signaling, tumor growth rates, and drug response depending on the system studied.

*In vivo* cell surface targeting is a complex interplay between tissue and imaging agent properties (Fig. 1). Investigators can manipulate the chemical or physical properties of the ligand by adding or removing functional groups to alter molecular weight, charge, affinity, and stability^2^,^3^,^4^. For example, molecular weight can be manipulated with recombinant proteins^5^,^6^ or through PEGylation of small molecules to slow blood clearance^2^,^7^. Plasma protein binding can also be manipulated through the net charge and charge distribution of small molecules^8^,^9^,^10^,^11^. With fluorescence imaging, even among water-soluble visible-light dyes, molecular charge and logD (pH 7.4) of the dye influence lipid bilayer interactions^10^. More recently, stabilization of peptide secondary structure has been used to increase cellular uptake and resistance to proteases^12^,^13^, which may also impact non-specific interactions. Equally important are the tissue properties and local physiology that vary widely between different tissues and in healthy versus diseased states^14^,^15^. Depending on the target tissue, intrinsic transport parameters such as blood flow (Q) and blood vessel surface area to volume (S/V) vary. The diverse local physiology results in tissue-specific optimal imaging agent properties, making it difficult to generalize from empirical rules or extrapolate from previous results. Likewise, computational approaches originally developed for therapeutics often require extensive experimental data or assume a pseudo-steady state drug concentration, which is not appropriate for imaging agent design. Many imaging agent models have been used to quantitatively analyze experimental data^16^, but most of these compartmental models were not designed to predict distribution based on probe properties. Therefore, imaging agents are typically developed on a case-by-case basis with low throughput and cost-intensive animal experiments. While a qualitative understanding of *in vivo* conditions offers insight on which parameters play a role in targeting, a quantitative model that is both mechanistic and predictive would allow for much more efficient design of imaging probes.

To help guide the design of novel imaging agents against extracellular targets, we present a quantitative and mechanistic model and *in vitro* experimental approaches for describing target tissue uptake and background signal. The simulations build on previously published models that incorporate the time-varying and spatially heterogeneous tissue concentrations present under non-equilibrium conditions following a bolus dose. The trade-offs in molecular properties can then be quantitatively compared for selection of the most promising agents to move forward with imaging probe development.

## Results

A simplified system was used to identify the most important parameters for maximizing contrast under optimal conditions. Assuming a high affinity binder with no blood flow limitations, first-order uptake in background tissues, and probe degradation rates that are slower than plasma clearance and internalization (SI), the maximum target-to-background ratio (TBR) can be expressed as


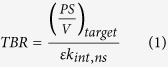


where P is the vascular permeability of the target tissue, S/V is the tissue vessel surface area, ε is the tissue interstitial void volume, and k_int,ns_ is the non-specific internalization rate in background tissue. Based on these results, the salient design parameters for targeting contrast are affinity, molecular weight (impacting P), stability/residualization of the label, and non-specific interactions. The optimal values of these parameters depend on the tissue blood flow, vessel-type, vessel surface area, target expression, and internalization rate.

Previous reports indicate that at molecular weights less than about 35 kDa, the efficiency of uptake in a tissue (% injected dose per gram) increases with reduced molecular weight due to increased permeability^4^. Eventually, the extraction fraction approaches 100%, providing an upper limit on the benefit of small size. Molecular weight cutoffs were established for optimal extraction of the imaging agent from the circulation to the target tissue. The extraction fraction is a function of molecular weight, plasma flow rate, effective permeability, and S/V^17^. The correlation between effective permeability as a function of molecular size was fit for fenestrated, non-fenestrated, and tumor vessels. As previously reported by Schmidt *et al.*^4^, tumor data indicated 4.5 and 500 nm radius pores with fractional area to thickness ratios of 17.6 and 0.65 cm^−1^ for small and large pores, respectively. Fenestrated vessels indicated 8 and 35 nm radius pores with fractional area to thickness ratios of 30 and 2 cm^−1^ for small and large pores, respectively. Non-fenestrated vessels suggested pore radii of 0.7 and 60 nm with fractional area to thickness ratios of 10 and 2 cm^−1^ for small and large pores, respectively. Using these permeability fits and a 50% extraction fraction cutoff, the prediction for tumor targeting indicates a molecule below 2200 Da will effectively extravasate out of the blood vessel into the tumor tissue (Fig. 2). Similar analyses are performed for organs and tissues with fenestrated or non-fenestrated vasculature. For tissue with non-fenestrated vessels, such as bone, heart, liver, and muscle, the molecular weight cutoff for 50% extraction is low due to high blood flow rates and low permeability: bone (<570 Da), heart (<100 Da), lung (<80 Da), muscle (<600 Da). Fenestrated organs resulted in higher molecular weight cutoffs as seen by kidney (<3600 Da), endocrine pancreas (<3000 Da), and exocrine pancreas (<6900 Da).

To examine the impact of affinity, we selected two series of compounds in this size range against a tumor antigen, HER2, and an endocrine pancreas target, GLP-1R, and compared experimentally measured versus predicted uptake. In the case of the high HER2 expressing tumor cell line (SKOV-3 > 10^6^ receptors/cell), predicted uptake approaches the maximum value of 14–15%ID/g at binding potential (BP = target concentration relative to dissociation constant, [T]/K_d_) values of 50 or greater (K_d_ < 20 nM) for these slowly internalized affibodies. Experimental results indicate affibody molecules with measured K_d_ < 20 nM indeed achieve 14–15%ID/g experimentally (e.g. PEP07127 with a 3.8 nM K_d_, Fig. 3B). For a tumor with ~30-fold lower expression (LS174T tumors = 30,000 receptors/cell), the affinity must be 30-fold higher for the same BP. Therefore, the K_d_ must be <0.7 nM for high uptake. In agreement with these results, PEP07127 has low uptake in LS174T xenografts, but ZHER2:342 (K_d_ = 0.022 nM) achieves high ~15% ID/g uptake.

The *in silico* predictions in most cases agreed with experimental results. These simulations assume complete residualization (all the internalized probe is trapped within the cell indefinitely). Metallic radionuclides such as technetium and indium are known to be residualizing labels^18^,^19^,^20^,^21^,^22^ over the time scale of the experiment, while halogen radionuclides such as iodine are non-residualizing^23^,^24^. This could explain the lower iodinated compound experimental values in Fig. 3B.

The predicted uptake in the endocrine pancreas is very different from the tumor in both magnitude and shape. The higher blood flow and permeability results in much larger maximum uptake values but also more stringent binding affinity requirements (a general shift to the right on the log scale of Fig. 3C). Because GLP-1R expression (54,000 receptors per cell) is closer to LS174T levels of HER2, a high affinity requirement would be expected. However, the internalization rate of GLP-1R is much faster (2 × 10^−3 ^s^−1^), trapping probe inside the cell before it can dissociate. Therefore, a K_d_ of only 10 nM is needed for efficient targeting. In agreement with these predictions, a decrease in affinity from 2.6 nM to 8.3 nM had a minimal impact on pancreatic uptake^25^.

To quantify the impact of background tissue signal, two additional probe data sets were examined – low molecular weight prostate specific membrane antigen (PSMA) ligands and integrin binding ligands. Plots of TBR versus blood clearance for both integrin and PSMA binders (Fig. 4A,B) show similar trends. In the case of PSMA binders, at 4 h post-injection, higher TBRs (tumor to muscle ratio, muscle uptake used as background) generally correspond with low blood concentration (MIP1095 excluded, circled). MIP1072 and MIP1404, the compounds with the highest 4 h TBR at 171 and 157 have the lowest blood concentrations at 0.06 and 0.02%ID/g, respectively. MIP1405, MIP1427, and MIP1428 have lower TBR values of 72, 31, and 42 respectively and displayed relatively slower blood clearance with 4 h blood values of 0.26, 0.28, and 0.14%ID/g. Integrin binders show a similar trend between TBR and blood signal (compound **17** omitted). These results indicate that in this size range (well below the renal filtration cut-off), more rapid plasma clearance benefits TBR by reducing the background. Although properties such as plasma protein binding may slow clearance and allow more time for target uptake, this effect is mitigated by the fact that plasma protein binding also reduces extravasation rates by reducing the concentration of unbound probe.

A series of three affibodies was studied by Tolmachev *et al.* to examine the impact of affinity and physicochemical properties of small proteins on targeting^26^. Comparing peptides PEP05838 and PEP05541, the sequence was more hydrophobic^27^ for PEP05838, resulting in higher liver uptake (Fig. 4C). The lowest uptake occurred for PEP07127, which notably differed by a hydrophilic residue in the middle of an otherwise hydrophobic patch on the surface. However, based on sequence alone, PEP07127 had an intermediate hydrophilicity, indicating secondary structure can play a driving role even for residue changes on a single alpha helix. Similar to integrin and PSMA binders, the blood clearance was compared to TBR (Fig. 4D), and the highest TBR had the lowest blood signal. Due to a wide range of K_d_ values for the 3 peptides in this analysis, the 4 h time points were evaluated to minimize the impact of affinity since at 24 h or with the low expressing cell line, the impact of affinity dominates over differences in non-specific interactions.

The data indicate that rapid plasma clearance is beneficial for molecular imaging agents in the single kDa range, and we hypothesized that non-specific interactions could quantitatively explain the differences in clearance from the blood in this size range. However, there is no simple correlation between physicochemical properties of small molecules or sequence of peptides and efficient clearance due to the large number of non-specific low affinity (ionic, hydrophobic, colloidal) interactions that are difficult to predict from structure alone. To prospectively explore the impact of physicochemical properties on clearance, a series of fluorescent integrin binding peptidomimetic agents was synthesized using different fluorescent dyes to manipulate the physicochemical properties. Two different assays were used to measure non-specific interactions – a non-specific cellular uptake assay and an equilibrium plasma protein binding assay. The cell-based assay used excess non-fluorescent probe to block specific interactions and measured the increase in fluorescence signal over time (presumably from non-specific surface protein and membrane interactions), and the plasma protein binding assay was used as a surrogate for overall non-specific interactions. Plasma clearance of the probes was measured in C57BL/6 mice over 24 h. The most hydrophobic agent had very rapid early phase decay but had higher signal at later times, while the more hydrophilic probes had increased clearance at 24 h (Fig. 5). Rapid redistribution into tissues lowers the plasma concentration initially, but whole body clearance from tissues has a larger impact on TBR^28^. Given the impact of peptide hydrophobicity on targeting and uptake, a series of fluorescent exendin peptides with varying hydrophobicity was also synthesized and administered intravenously to mice. The non-specific cellular uptake rates of the molecules were plotted as a function of the plasma concentration following IV delivery and demonstrated a strong correlation between plasma clearance and non-specific interactions (Fig. 5F). Both the non-specific cellular uptake rate and plasma protein binding correlated with plasma clearance and can be run in a high-throughput manner. Each method had different strengths. The plasma protein binding assay is cell-free, resulting in less variability, and protein binding also affects uptake in the target tissue^29^, so it is useful for correlating with both uptake and background signal. However, for certain compounds it is difficult to achieve equilibrium using dialysis. In these cases, such as with the exendin peptides, proteins, and compounds that interact strongly with the dialysis membrane, the cellular uptake assay could be used.

Given the increasing use of near-infrared (NIR) fluorescence in preclinical studies and clinical applications (for intraoperative imaging), we measured the protein binding for a series of fluorescent dyes (Table 1). While the targeting ligand and linker also impact the overall plasma protein binding, the relative scale of these values is useful in estimating overall probe PPB.

## Discussion

Molecular imaging agents can provide detailed insight into *in vivo* biological phenomena in a minimally invasive manner. However, the development of these agents is time consuming and costly. There are often tissue and target-dependent trade-offs between properties that maximize signal, foiling empirical approaches and extrapolation from published values. To improve the design of imaging agents and help focus preclinical experiments on the most promising molecules, we analyzed and quantified the mechanistic trade-offs for probe development.

**Figure 1:**
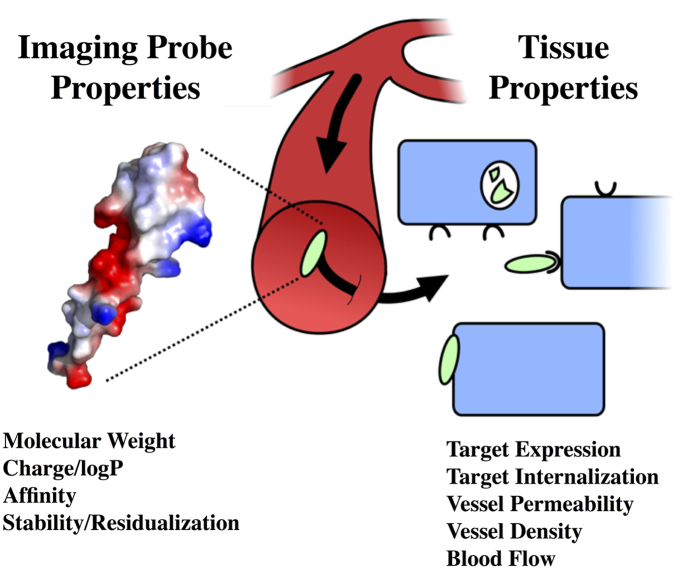
Diagram of the interplay between imaging probe properties and target tissue properties in determining contrast. Exendin is shown with PyMol surface charge as the sample imaging agent (PDB 1JRJ).

Parameters that determine target uptake for *in vivo* imaging can be categorized as either an imaging probe property or a tissue property (Fig. 1). Molecular weight and charge play a role in uptake and clearance, while affinity and label residualization impact target retention. These imaging agent values are all relative to the tissue properties, making generalizations difficult. For radionuclides, the choice of radioisotope and chelator are also critically important. Certain aspects such as ease of synthesis and stability of the complex *in vivo* are beyond the scope of this analysis. However, other properties, such as the radioisotope decay half-life (where isotopes with short half-lives are acceptable for rapidly cleared agents), residualization rate of the radioisotope, and chelation chemistry (which impacts plasma protein binding and non-specific interactions) all have an impact on the parameters discussed here.

The molecular properties of the targeting probe are under the direct control of the researcher, and these must be optimized according to the target tissue type for maximum signal and contrast. Based on a previous analysis of targeting efficiency in tumors, two local maxima exist for target uptake – one at single kDa molecular weight ranges and one close to the size of antibodies^4^. For imaging agents, clearance is important for contrast, so lower molecular weight probes result in higher target to background ratios. Antibodies rarely have tumor to organ ratios above 100 and are typically 3–10^30^,^31^. However, several low molecular weight probes cited in this work reach ratios above 100, and importantly, these high TBR values developed within several hours after injection versus days for antibodies. Therefore, we focused on the optimization of low molecular weight compounds in this analysis.

Based on size versus permeability and clearance, a lower molecular weight is more efficient in target uptake. However, we sought to determine the optimal size of an agent, since eventually blood flow limitations negate any benefits from smaller size. Molecular weight cutoffs were established for various tissues using a 50% extraction fraction criterion (related to the vessel depletion number^17^). Predictions indicate non-fenestrated organs with small pore sizes such as bone, heart, lung, and muscle have a significantly lower size cutoff compared to the fenestrated kidney and pancreas. The predictions also indicate that in organs with high plasma flow such as heart (4.06 mL/min/g) and lung (24 mL/min/g), the extraction fraction can be very low for many probes (albeit a low fraction of extremely high delivery).

The blood flow and extraction fraction determine the amount of probe reaching the tissue, but this does not account for binding and retention of the imaging agent at the target. While antibodies can readily be engineered for high affinity (~1 nM K_d_), high affinity low molecular weight compounds can be more challenging to design^32^,^33^,^34^,^35^. Given this common trade-off between molecular weight and affinity (such as dimerization increasing apparent affinity but also molecular weight^36^), we next determined the required dissociation constant for efficient localization.

A predictive and mechanistic model was used for comparison with published values of tumor and endocrine pancreas uptake for two classes of targeting molecules (Fig. 3) – HER2 binding affibodies (MW ~ 6 kDa) and GLP-1R binding peptides (MW ~ 5 kDa for monomer). Tumor cell lines with high (SKOV-3) and low (LS174T) expression of HER2 were simulated for the HER2 agents. To account for varying expression levels, the binding potential was compared to the internalization rate. Varying either [T] or K_d_ to manipulate BP gave the same results. Predicted uptake values were calculated assuming tracer (subsaturating) doses. However, tumor xenografts with low expression levels can readily be saturated, and several data points were excluded due to reported evidence of target saturation^37^. Because plasma protein interactions are not well-characterized for these molecules in the literature, a single value of plasma protein binding was assumed (42% bound); this is the experimentally estimated value for fluorescent exendin, a peptide with similar molecular weight and alpha helical secondary structure. However, the value could be higher due to possible interactions with other plasma proteins^38^.

**Figure 2:**
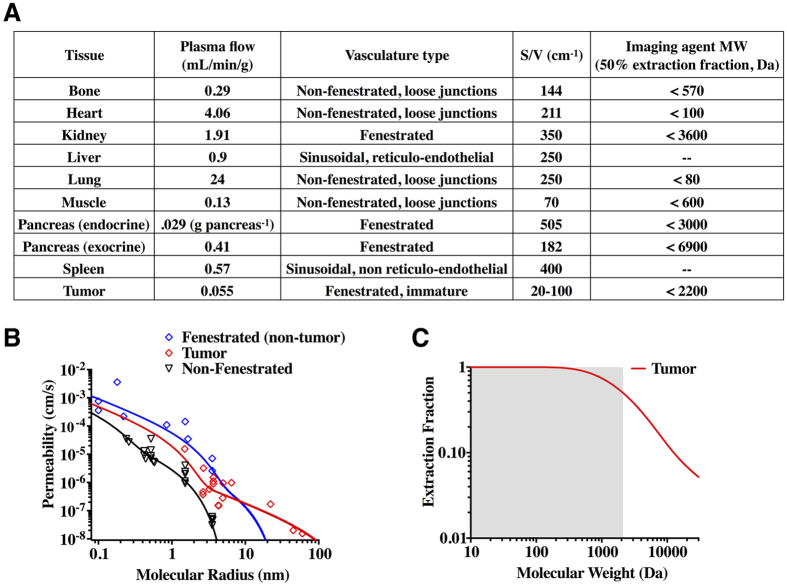
Tissue Physiology versus Molecular Weight: the optimal molecular weight probes for different tissues. The values are based on 50% extraction fraction using the vessel type, blood flow, and vessel surface area (**A**). Tumors have elevated macromolecular permeability, but the elevated interstitial pressure may contribute to lower effective permeability than fenestrated tissue for low molecular weight compounds. The data and fit for tumor values are identical to ref. 4 (**B**). The 50% extraction fraction is an arbitrary cutoff based on diminishing returns as seen for the tumor (**C**).

The simulation results agreed well with the literature reported values for HER2 (Fig. 2B). With the very high expression levels in SKOV-3 cells, a 3.8 nM binder (PEP07127) is sufficient for high uptake, but a 50 nM K_d_ (ZHER2:4) lowers the signal. In contrast, the lower expression on LS174T xenografts results in lower uptake of the 3.8 nM binder and requires a 22 pM affinity (ZHER2:342) for high retention. (Note that the required affinity is also a function of the molecular weight, and for low affinity interactions, higher targeting efficiency can often be achieved by increasing the molecular weight above the optimal range discussed here^4^.)

The model predictions are also in agreement with uptake of exendin monomers, dimers, and trimers in the endocrine pancreas despite very different tissue physiology. For the slowly internalized affibody-HER2 complex^39^,^40^ (3.9 × 10^−6^ s^−1^), a very high affinity (<1 nM) was required for maximum uptake with moderate receptor expression (LS174T). In contrast, the much more rapidly internalized GLP-1 receptor (2 × 10^−3^ s^−1^) has a lower requirement (<10 nM) for high uptake even with similar expression levels as LS174T (54,000 GLP-1 receptors versus 30,000 HER2 receptors per cell) as seen with the 8.3 nM binder. Uptake is only noticeably reduced with a K_d_ of 20.9 nM. The model slightly over-predicts uptake, which could be a function of error in any of the model parameters that were not fit, such as plasma protein binding, wt% of beta cells, permeability, etc. Importantly, these simulations highlight the differences in affinity requirements based on tissue properties and target internalization. Since these probes are given at tracer doses, internalization does not amplify the signal like it would for a saturating dose. However, it does reduce the required affinity since the probe is internalized and trapped prior to dissociation. These data demonstrate that the required affinity is a function of target expression, tissue physiology, and internalization rates.

Unlike target uptake rates that can be predicted with reasonable accuracy from sigmoidal binding curves and measured extravasation rates, the background signal is more challenging to predict. Low molecular weight agents are cleared more rapidly than higher molecular weight agents^41^,^42^, but for small molecules well below the nominal renal molecular weight cut-off, non-specific interactions play a large role. For these agents, molecules that interact strongly with plasma proteins will have slower clearance times^29^,^43^. While this may allow more time for uptake, this also reduces extravasation in the target tissue^29^, resulting in little to no net gain. In addition, non-specific interactions can increase background signal.

Although there may be no net gain in target uptake relative to plasma protein binding, we hypothesized that molecules with weak plasma protein interactions and fast blood clearance times would have lower background uptake *without a loss* in target uptake, resulting in higher TBR when imaging. This is in contrast to known trends for larger proteins where lower molecular weight results in a higher TBR but at the expense of lower target uptake^5^. Importantly, plasma protein binding can be measured in a high-throughput manner in contrast to animal biodistribution experiments, thereby providing a screening tool. To demonstrate this trend, two published series of low molecular weight targeting ligands with varying affinity and clearance rates targeting either PSMA or integrin of the form α_v_β_3_ were considered. At long times post-injection, both sets of molecules showed that the highest TBR values were obtained with the lowest blood %ID/g (Fig. 4A,B). There was also a general correlation with higher TBR with lower blood signal, although the data are scattered due to the many additional factors that impact TBR beyond background clearance (e.g. affinity), and two outliers are discussed in more detail. One outlier in the PSMA set (Fig. 4A dotted circle) is compound MIP1095, which exhibited both slow clearance and a high TBR. This molecule exhibited the strongest affinity for all PSMA binders tested (K_d_ = 240 pM), the highest tumor uptake (34.3%ID/g), and the highest skeletal muscle uptake (0.26%ID/g). This outlier demonstrates one of many quantitative trade-offs, where having a strong affinity at the cost of poor clearance can still result in a high TBR. All properties (not just plasma clearance) must be quantitatively considered together.

The nine bivalent integrin-targeting agents designed with various spacer lengths also support the notion that molecules that have a low blood concentration at 24 h post-injection are able to achieve a higher TBR. Again, the highest TBR values corresponded to the lowest blood signal. One outlier is compound **17** (Fig. 4B dotted circle), where both blood concentrations and TBR at 24 h were low at 0.031%ID/g and 18, respectively. However, this results from unusually high muscle uptake of >0.6%ID/g - much higher than all the other compounds in the series and actually increasing from 4 to 24 h. This suggests specific uptake of the probe in muscle or an experimental artifact.

Weak and non-specific interactions between molecules are challenging to predict. These interactions are being investigated for large proteins such as antibodies, and comparisons with these findings provide some insight^44^,^45^. Hydrophobic and charged patches on the surface of these proteins can increase colloidal interactions, changing the pharmacokinetic properties. Notably in the case of antibodies, non-specific tissue interactions *increase* clearance from the normally long-circulating molecules^46^, while plasma protein interactions typically *decrease* the clearance of the normally rapidly-cleared small hydrophilic molecules discussed here.

PSMA and integrin ligand results show there is no simple correlation between TBR and overall charge (SI). Many of the charges come from the chelating agent, and chelator selection is a critical component of probe design^47^,^48^. Some chelated metals have very low non-specific interactions like indium-DTPA while others have higher plasma protein binding and overall lipophilic properties like Tc-99m-DTPA^28^,^49^. More hydrophilic chelators have been designed, but here again these do not completely correlate with physicochemical properties^50^, making predictions from structure alone challenging.

Tolmachev and colleagues’ analysis of non-target uptake for affibodies as a function of hydrophobicity provides insight into peptide agent design compared to non-peptide small molecule ligands^26^. Compound PEP07127, due to an L18S amino acid substitution, disrupted a hydrophobic patch present in the original compound. Even for this small region on an alpha helix, the secondary structure played a role in non-specific interactions. As pointed out by these authors, the results are pertinent to the development of high affinity proteins and peptides, where many binding interfaces have hydrophobic regions driving high affinity^26^, highlighting a direct trade-off between affinity and background signal. Hackel *et al.* investigated the impact of surface charge and hydrophilicity modification on fibronectin compounds^51^. By manipulating the surface properties, they were able to control the distribution in the two major clearance organs, liver and kidney. Our current analysis does not focus on clearance organs, but for the integrin and PSMA binders, more efficient clearance from the blood generally correlated with lower signal in the liver and kidneys for these non-peptide agents.

The affibody results highlight the fact that predicting the impact of amino acid substitutions on the abundant *in vivo* low affinity interactions that depend on sequence and structure is extremely complex and challenging, particularly in the context of screening compounds where the structure may not be known. Rather, taking a lead from the protein engineering field^44^,^45^,^52^, experimental methods of measuring non-specific interactions in a high-throughput manner may be more fruitful. Hydrophobic and other non-specific interactions impact off-target uptake of these molecules (Fig. 4C) and directly lower tumor to organ ratios. Conversely, the highest TBR value occurred for the compound with the lowest blood signal, consistent with the integrin and PSMA imaging agent series. Slow clearance is likely a direct result of increased plasma protein interactions, which is challenging to quantify for peptides. Accurate plasma protein quantification for lower molecular weight molecules is possible using several techniques, such as rapid equilibrium dialysis or ultrafiltration, and these were investigated next.

To compare two methods of measuring non-specific interactions for correlating with plasma clearance, we generated a series of small molecule fluorescent peptidomimetics using different fluorescent dye structures to manipulate the physicochemical properties. Non-specific interactions due to lipophilicity have been characterized for many visible light fluorophores^10^,^11^ but less is known about these interactions for near infrared (NIR) fluorophores, which have *in vivo* advantages for imaging. *In vivo* clearance and non-specific uptake rates of peptidomimetics using NIR fluorophores such as AF680, 800CW, and ZW800 demonstrate the impact of non-specific interactions on imaging agent design. The plasma clearance was measured over 24 h (Fig. 5A,B) and compared to plasma protein binding measured by rapid equilibrium dialysis and non-specific uptake in cell culture. The cell uptake rates correlated with plasma protein binding, indicating these assays may be providing similar information on non-specific interactions (Fig. 5C). The probes with lower plasma protein binding typically had more rapid alpha phase clearance and lower plasma signal at 24 h. Given the correlation with higher TBR in Fig. 4, this could be incorporated into high throughput screening methods for determining propensity to clear from tissue. Note that the most lipophilic agent (BODIPY 650 conjugate) had a fast early (alpha phase) clearance but higher signal at 24 h. It is important to distinguish rapid clearance from rapid redistribution of probe from plasma to tissue, since the former can lower background signal while the latter can significantly increase it (e.g. PEP05838, Fig. 4C^26^).

The plasma protein binding assay has the advantage of a cell free system (reduced variability) and simultaneously provides data on target uptake (since it is generally unbound probe that enters tissue). However, commercially available assays typically only work for probes less than 1–2 kDa in size and can have problems with agents that have extensive interactions with the dialysis membrane, making the cell uptake assay a viable alternative. As an example, four exendin peptide conjugates were synthesized with varying hydrophobic properties. Although plasma protein could not be quantified due to their higher molecular weight, the non-specific cell uptake rate was significantly higher for the more lipophilic conjugates and correlated with the 3 h plasma concentration in mice (Fig. 5F).

The relative values of plasma protein binding and non-specific cell uptake can be used to rank compounds, but an absolute guideline is helpful in determining if further molecular engineering is warranted. Similar to reductions in molecular weight and increases in affinity, there are benefits of increasing hydrophilicity to reduce background (such as addition of PEG to integrin binders^53^), but eventually there are diminishing returns with further effort. Based on the data presented here and literature values (SI), a significant reduction in background is not expected below 60–80% plasma protein binding or 10^−5^ s^−1^ cellular uptake rate. (For comparison, pinocytosis rates^54^ have been estimated around 1.1 × 10^−5^ s^−1^.) Further improvements beyond these levels may be obtained by reducing these parameters, but this is not without cost. Molecules in this size range that have no measurable plasma protein binding, inulin being a classic example, are very hydrophilic and flexible in direct contrast to most high affinity binding interfaces.

Given the increasing use of near-infrared probes for preclinical studies and clinical intraoperative imaging, we quantified the plasma protein binding of several commercially available dyes to guide fluorescent probe development. While the conjugates of these dyes will have altered plasma protein binding (and lose the carboxylic acid once they are conjugated to an amine), the relative magnitude can be used to estimate overall properties. For dyes in the 680–700 nm range, Alexa Fluor 680 had the lowest plasma protein binding of the dyes tested, while for the 750–800 nm range, ZW800-1 had the lowest plasma protein binding. These values, in addition to the dye residualization rates^55^, are helpful in rational design of NIR probes.

**Figure 3:**
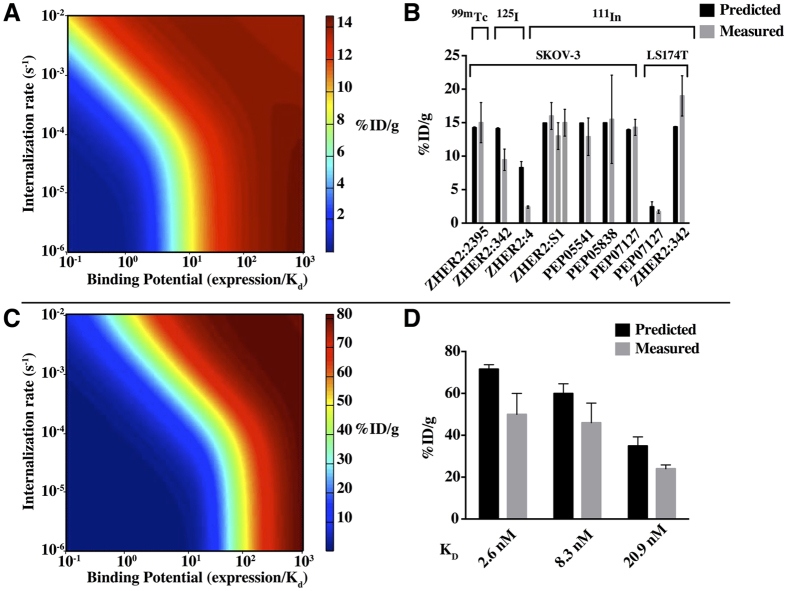
A computational model of tumor and endocrine pancreas targeting with peptide agents versus experimental results. Using the physiology of a tumor, the targeting efficiency (%ID/g) is graphed versus the internalization rate and binding potential (a function of target expression and affinity, (**A**)). These results are compared with a series of affibody molecules varying in affinity, radiolabel, and target expression (**B**). The physiology of pancreatic islets results in much higher potential targeting %ID/g due to higher blood flow and vascular density. The more efficient transport also shifts the contour lines to the right, requiring higher affinity due to faster washout (**C**). A comparison of predictions with published data on exendin based peptides of varying molecular weight and affinity highlights how the rapid internalization reduces the stringency in binding affinity despite the faster washout (**D**).

**Figure 4:**
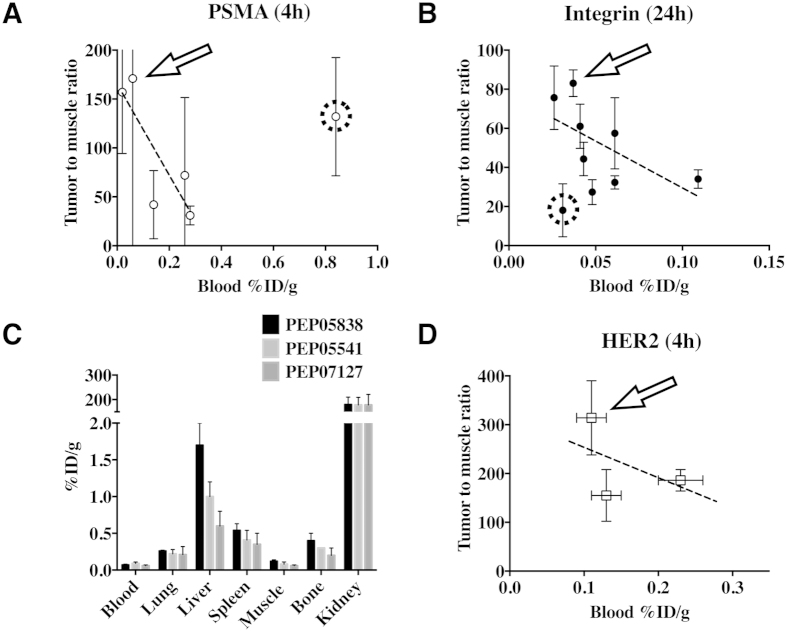
Impact of non-specific uptake in background tissue: a comparison of blood clearance to TBR. For both low molecular weight PSMA binders (**A**) and integrin binders (**B**), the highest TBR values (arrows) occur for low blood signal. The lines indicate a general trend of lower TBR with higher blood signal. The circled data were excluded as described in the text. Although faster clearance has the potential to lower target uptake and reduce TBR, these results indicate this is generally not the case for probes in this size range. For proteins/peptides, even a single alpha helix can result in secondary structure playing a significant role in determining non-specific uptake (**C**). The substitution of a single amino acid in the middle of a hydrophobic patch on PEP07127 reduced background tissue concentrations, resulting in the highest TBR and fastest blood clearance (**D**). Data adapted from Tolmachev *et al.*^26^.

**Figure 5:**
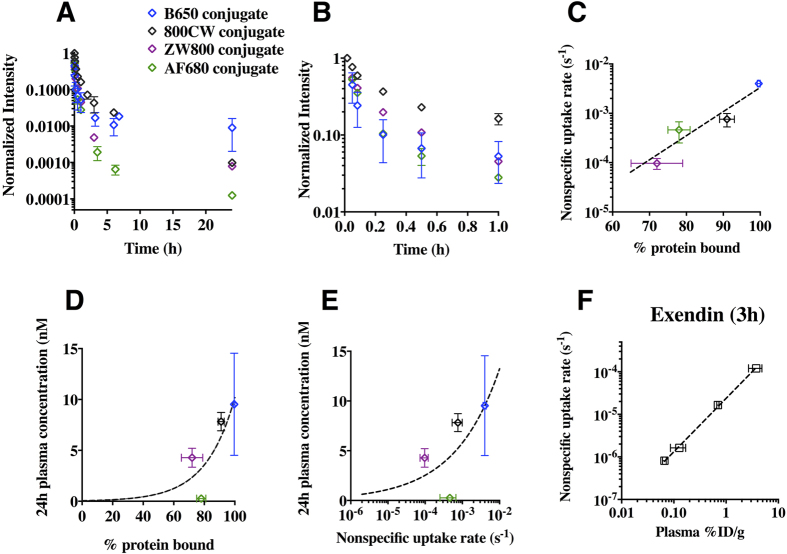
High throughput methods for quantifying non-specific uptake. Lower plasma protein binding and non-specific cell uptake generally correlate with faster plasma clearance for a series of integrin probes with the exception that very lipophilic compounds can redistribute rapidly in the tissue, causing high background but low plasma signal (**A**) particularly over the first hour (e.g. BODIPY 650 conjugate, (**B**)). Plasma protein binding, a common assay for small molecule drugs, correlates with non-specific cell uptake *in vitro* (**C**). For plasma protein binding, there are diminishing returns when lowering the fraction below ~60% bound (**D**). Based on the non-specific cell uptake assay, which is useful for agents that cannot undergo rapid equilibrium dialysis (e.g. due to molecular weight or membrane sticking), a non-specific *in vitro* cell uptake rate of less than ~10^−5^ s^−1^ also has diminishing returns (**E**) providing quantitative guidance on probe selection. Four exendin derivatives where plasma protein binding could not be measured directly show that the non-specific cell uptake assay correlates with plasma clearance (**F**).

In conclusion, we have analyzed data from 32 low molecular weight probes to provide a quantitative and mechanistic framework for comparing cell surface imaging agents. The qualities of optimal imaging agents are well known, but in practice, there are many trade-offs. This analysis is designed to quantitatively determine the magnitude of these impacts to select the most promising agents for preclinical analysis. For example, data on blood flow and permeability could be used to identify an appropriate scaffold and/or library for screening based on the target tissue (Fig. 2). The internalization and expression level of the target can be used to define a target affinity or compare predicted retention of lead compounds (Fig. 3). A high throughput test of plasma protein binding and/or non-specific cellular uptake can be used to quantitatively compare liabilities in tissue background signal (Figs 4 and 5). Alternatively, for a series of *in vitro* characterized compounds, these plots can be used to quantitatively compare the expected TBR based on molecular weight, affinity, and lipophilicity/non-specific interactions. These data will help select the most promising candidates to move forward in a more quantitative and rational manner for molecular imaging agent design.

## Methods

### Computational Model Development

The original tissue simulation model was based on a non-linear partial differential equation model of tissue concentration with time-varying mixed boundary conditions and axial and radial gradients^56^. This model provides a self-consistent framework for simulating the distribution of agents from small molecule drugs to biologics and nanoparticles^57^. To limit the scope of the simulations, we used previous modeling results indicating that the highest target uptake occurs for very low molecular weight agents and those similar in size to antibodies^4^. In addition to high uptake, low molecular weight compounds benefit from rapid clearance and have demonstrated target to muscle ratios over 100^58^,^59^. To focus on the design of imaging agents for extracellular targets, lipophilic compounds that can diffuse across plasma membranes with significant retained intracellular signal were excluded^60^. Finally, since imaging agents are ideally delivered at tracer doses, a subsaturating dose was assumed. Based on the four classes of pharmacokinetic behavior in tissue (blood flow-limited, permeability-limited, diffusion-limited, or binding/metabolism-limited agents)^57^, the lack of transcellular extravasation of highly lipophilic compounds and tracer dose eliminates the diffusion-limited and binding/metabolism-limited regimes, respectively, narrowing the analysis to blood-flow and permeability limited probes. Since peptide and protein-based agents form a large contingent of extracellular imaging agents, we focused on a permeability-limited model subject to blood-flow limitations.

A compartmental analysis assuming equilibrium binding was performed on plasma, tissue, and internal compartments (SI). Briefly, the imaging agent extravasates from the plasma compartment into the tissue compartment, where it is either receptor-bound or free. Bound imaging agent is then trafficked to the internal compartment. Binding equilibrium is assumed to be very fast relative to diffusion of the imaging agent out of the plasma. The model also assumes a sub-saturating tracer dose of imaging agent with the receptor in excess. It can be shown (SI) that the concentration of imaging agent in the extracellular tissue and internal compartments as a function of time can be represented by


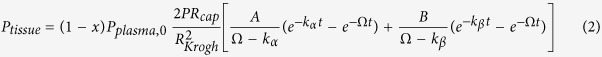


and


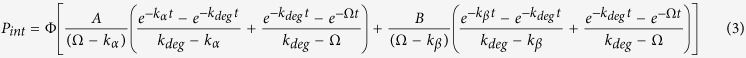


where


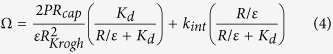



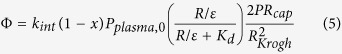


The parameters are described in the supplementary file. This model was used in conjunction with experimental data and simplifying assumptions to quantify the impact of probe and tissue parameters on target to background signal. While high contrast is not always necessary for an imaging agent, in most cases (delineating diseased tissue, quantifying changes in receptor expression) high contrast is beneficial and reduces artifacts from surrounding tissue signal.

The strategy outlined in this approach aims to maximize TBR by internalizing the probe within the target cells and minimizing binding/sticking and internalization in non-target tissues. An alternative approach is to select targets that are slowly internalizing and design probes that rapidly leak out of cells once they are metabolized. This would lower the signal intensity in off-target and clearance organs such as the liver and kidney. While this has been shown to improve target to background ratios for these organs^61^, there are several limitations to this approach. The internalization, metabolism, and clearance of the probe from non-target tissue has to occur much faster than in the target tissue. Since metabolism and clearance often takes several hours to a day or more, ideally the target would not be internalized significantly over this time frame. Because most cell surface proteins have a constitutive internalization rate of several hours^62^, this greatly limits the number of applicable targets. As noted in Fig. 3, this can increase the required affinity for high target uptake by 10 to 100-fold. Finally, the lipophilic chelate can increase plasma protein binding and liver uptake, lowering targeting efficiency. Although this approach can decrease the liver signal more than it decreases the target signal^61^, in contrast, the approach discussed in the current work decreases non-specific uptake while *increasing* target signal. This may explain why the reported TBR values for the current approach are much higher than those achieved by a non-residualizing agent.

### Permeability versus Molecular Weight

The relationship between molecular radius and permeability was modeled using a two-pore representation as described by Schmidt *et al*^4^. Previously reported measurements for permeability (P) for small molecules, peptides, proteins, polymers, and liposomes were compiled from literature (SI) and fit to determine the radii and fractional area of small and large pores. To quantify the extraction ratio of various molecular weight molecules, the permeability was first calculated using the two-pore prediction with the appropriate tissue vasculature. Then, using experimentally measured values for plasma flow rate and S/V (SI), a mixed flow and permeability-limited model was adopted from Tofts *et al.* to predict the fraction of imaging agent extracted from blood to tissue^17^. Organ systems were categorized as either fenestrated (kidney, pancreas), non-fenestrated (bone, heart, lung, muscle), or tumor for analysis (SI). A two-pore model was used to generate optimized fits for each of the three categories based on vasculature type.

### Affinity versus Expression and Internalization

To predict uptake in various tissue types, a previously published model was used^56^. In short, the non-linear partial differential equation (PDE) model utilizes a Krogh cylinder geometry of tissue vessels with both axial and radial gradients for the imaging agent. Plasma concentration is modeled with biexponential decay with local concentrations determined by blood velocity and vessel permeability. A list of parameters used for tumor and endocrine pancreas uptake predictions are found in the supplementary data (SI). Experimentally measured affinity values were adjusted for temperature assuming constant free energy of binding. MATLAB was used to solve the system of nonlinear PDEs using finite differences to quantify tissue uptake. To generate a full %ID/g contour, internalization rates were varied between 10^−6 ^s^−1^ to 10^−2 ^s^−1^. Fixing antigen concentration and varying affinity generated a range of binding potential from 10^−1^ to 1000. Identical results were obtained when varying the binding potential by fixing affinity and varying the antigen concentration. Predicted uptake values were compared with experimentally determined values reported in the literature. For endocrine pancreas simulations, whole organ %ID/g was converted to islet %ID/g assuming islets are 1 wt% of the pancreas. Uptake values with 10% increases and decreases in internalization rate, affinity, and antigen expression were used as simulation error.

### Plasma Clearance versus Contrast

The impact of plasma clearance on TBR was investigated using previously published data from mammary and prostate tumor models (SI). In short, small molecule inhibitors targeting prostate-specific membrane antigen (PSMA) with various dissociation constants and blood clearance rates were used to target LNCaP or PC3 xenografts in mouse models. Measured values for tumor and skeletal muscle uptake were used to calculate the TBR, which was then plotted against the blood concentration. A similar analysis was performed for various integrin binders targeting mammary adenocarcinomas in a mouse model and HER2-binding affibodies. TBR for both sets of compounds were also plotted against the total compound charge at physiological pH to demonstrate a lack of trend between uptake and molecular charge (SI). An analysis of non-target uptake was performed as well for molecules of varying hydrophobicity using previously published data of anti-HER2 affibodies (SI). Additionally, a correlation between plasma clearance and non-specific interactions was explored for exendin, a 4 kDa therapeutic peptide used to treat type 2 diabetes. The hydrophilic dye Alexa Fluor 680 or a lipophilic non-sulfonated Cy7 dye were conjugated to exendin^25^ using either directly to the 14^th^ position or using a helix-stabilizing linker^12^ to generate four variants. The peptides were administered intravenously in C57BL/6 mice and the plasma concentration monitored over time. Non-specific cellular uptake for peptide conjugates in HT1080 cells was quantified as well using *in vitro* assays (SI).

### Plasma Clearance versus Non-specific Interactions

The targeting ligand was synthesized by ChemPartner (Waltham, MA) as an ester. The ester was hydrolyzed with 150 μL of ethanol and 7 μL of 1 M NaOH per mg of drug overnight, neutralized with 1 M HCl, and dried under vacuum. IRDye 800CW NHS ester was obtained from LI-COR (Lincoln, NE), ZW800-1 NHS ester was obtained from Curadel ResVet Imaging (Worcester, MA), and Alexa Fluor 680 NHS ester and BODIPY 650/665-X NHS ester were obtained from Life Technologies (Carlsbad, CA). The hydrolyzed integrin binder was reacted with the fluorescent dyes in a 1:1.5 molar ratio in the presence of 2 μL of triethylamine per mg of drug overnight and purified using a preparative scale Luna C18(2) column (Phenomenex; Torrance, CA) on a Shimadzu reversed phase HPLC unit. Successful conjugation was confirmed by ESI-MS and MALDI-TOF (SI).

All animal experiments were approved by the University of Michigan Institutional Animal Care and Use Committee (IACUC) and carried out in accordance with their guidelines. To quantify the blood clearance, the fluorescent compounds (15 nmol) were injected via the tail vein in C57BL/6 mice. Blood samples were collected at predetermined time points and the plasma concentration of the compounds was quantified using an Odyssey CLx. To quantify non-specific uptake rates, MDA-MB-231 cells were plated in 96 well plates and allowed to attach overnight. The cells were incubated with 200 nM of non-fluorescent ligand for 15 minutes followed by a co-incubation with 10 nM or 20 nM of fluorescent ligand and 100 nM of non-fluorescent probe. At 15, 30, 45, and 60 min, the cellular uptake was quantified using an Attune Acoustic Focusing Cytometer (Applied Biosystems) with fluorescent beads (Bangs Laboratories, Inc.) to measure the non-specific uptake rate. Plasma protein interactions were quantified for commonly used, commercially available far-red and near-infrared fluorescent dyes. For IRDye 800CW, ZW800, BODIPY 650, Alexa Fluor 680, and integrin conjugates, a Rapid Equilibrium Dialysis (RED) plate (Thermo Scientific) was used following the manufacturer’s protocol.

## Additional Information

**How to cite this article**: Zhang, L. *et al.* Mechanistic and quantitative insight into cell surface targeted molecular imaging agent design. *Sci. Rep.* **6**, 25424; doi: 10.1038/srep25424 (2016).

## Supplementary Material

Supplementary Information

## Figures and Tables

**Table 1:** Plasma protein binding of near-infrared dyes and conjugates.

Compound	Ex/Em (nm)	% Plasma Protein Bound
BODIPY 650 conjugate	646/660	99.6 ± 0.4
Alexa Fluor 680 conjugate	684/707	78 ± 3
IRDye 800CW conjugate	774/789	91 ± 2
ZW800 conjugate	768/786	72 ± 7
BODIPY 650 carboxylate	646/660	99.8 ± 0.3
Alexa Fluor 680 carboxylate	684/707	81 ± 6
IRDye 800CW carboxylate	774/789	89 ± 3
ZW800-1 carboxylate	768/786	81 ± 5*
ICG dye	~800/826	99.5–99.975*
SIDAG dye	755/778	57*
Alexa Fluor 647 carboxylate	651/672	76 ± 1
DDAO carboxylate	646/659	98.8 ± 0.2

^*^ZW800 carboxylate protein binding results displayed higher variation than other compounds (SI). ICG and SIDAG protein binding discussed in SI.
