# Supplementary material for: Mechanistic and quantitative insight into cell surface targeted molecular imaging agent design
Source: Sci Rep. 2016 May 5;6:25424. doi: 10.1038/srep25424 (PMC4857130; doi:10.1038/srep25424)
Supplement: Supplementary Information [file srep25424-s1.pdf]

Supplementary information

Mechanistic and quantitative insight into cell surface targeted molecular imaging agent design

Liang Zhang[*§], Sumit Bhatnagar[*§], Emily Deschenes[*], and Greg M. Thurber[*#]

[*] Department of Chemical Engineering, University of Michigan, Ann Arbor, MI 48109
[#] Department of Biomedical Engineering, University of Michigan, Ann Arbor, MI 48109
[§] These authors contributed equally to this work.

Table of Contents

1. Literature data on plasma protein binding cutoff
2. Reported plasma protein binding data and cell uptake
3. Modeling simplification and analysis
4. Capillary permeability data
5. Simulation values
6. Impact of vascular density on simulations
7. Probe synthesis and characterization
8. Charge versus Tumor to Muscle Ratio Plot
9. Exendin non-specific uptake

1. Literature Data on Plasma Protein Binding Cutoff

The 60% cutoff includes other examples from the literature of low plasma protein binding molecules in addition to the integrin binders presented here. Not many papers report the plasma signal at 24 h for low molecular weight compounds with low plasma protein binding, likely because the sensitivity of the technique has to be very high to detect several logs of clearance. It is also pertinent that extrapolation of a bi-exponential decay is not appropriate. While this provides a good description of clearance over several hours, over longer times other mechanisms besides redistribution and liver/kidney clearance become important. For example, the data in Fig 5A was not fit to a bi-exponential decay, since these fits could not account for the 24 h time point. Although it does not have a functional handle to label molecules, the NIR dye SIDAG has a reported low 57% bound fraction in the plasma. This uses two sugar molecules to provide hydrophilicity along with two sulfate groups, resulting in a hydrophilic dye with only -1 net charge. With SIDAG, the authors postulated that tubular reabsorption from the kidney as a mechanism that could play a role in the signal over 24 to 48 hrs. Since the clearance values are several orders of magnitude (3-5 logs of clearance), even a small amount of reabsorption could impact the plasma signal.

Harris et al. 2003 reports a 24 h blood signal of 0.06 %ID/g[1]. Estimating the initial signal as ~31 %ID/g (100% of the dose in 3.2 mL of blood assuming the reported 27 g mouse[2] and 12 mL/100g[3]), this would be approximately 3 logs of clearance. Although not specified in the thesis, if this molecule is the same as ^{111}In-TA138 (based on identical images in Fig. 3.2 of the thesis and Fig 5 of Harris et al.), then the measured plasma protein binding *in vivo* was 28.4% +/- 8.3% bound.

Another estimate of plasma clearance for a low molecular weight compound can be found in the pre-targeting literature. Kranenborg et al. reported a 24 h time point for tumor and the tumor-to-blood ratio with In-DTPA. Using the reported tumor signal at 24 h (1.3 %ID/g) and the tumor-to-blood ratio of 480, this results in a blood signal of 0.0027 %ID/g at 24 h. Using the same initial concentration in the blood as above (31 %ID/g), this results in approximately 4 logs of clearance. Since In-DTPA has less plasma protein binding than 99mTc-DTPA[4], which has been measured at < 5%[5], the protein binding is negligible for In-DTPA. One caveat of this estimate is that 4 days prior to the measurement, a bispecific antibody was injected, which could slow down clearance due to specific binding. However, the In-DTPA was given in 10-fold excess, and based on the reversible monovalent binding to the antibody, it is not known how much this impacts the 24 h clearance.

The two results above with much lower plasma protein binding levels than the current dyes indicate that 3-4 logs of clearance may be the maximum attainable. Since 3-4 logs of clearance were obtained with the AF680 and ZW800 conjugates that have 70-80% plasma protein binding, there is likely a limited benefit in obtaining a lower fraction bound. Based on the extrapolation in Fig. 5C, the limit of 60% bound was selected.

2. Reported plasma protein binding data and cell uptake

Rapid Equilibrium Dialysis (RED) was used to quantify plasma protein binding for various fluorescent dyes (carboxylate form) and fluorescent conjugates. RED plates are available with membrane molecular weight cutoffs of either 8 kDa or 12 kDa. Given that many of the fluorescent conjugates are >1 kDa, the 12 kDa plate were used to reduce the equilibration time between chambers (Table 1). The 12 kDa RED plate gave consistent results with small variation for all compounds tested except for ZW800 carboxylate, as seen in the higher standard deviations reported. It is possible the overall net positive charge on the molecule is responsible for interactions with the dialysis membrane. The 8 kDa RED plate resulted in lower plasma protein binding, which would be unexpected based simply on equilibration time. An ultrafiltration method (Centrifree Ultrafiltration Device, Millipore) resulted in lower but variable values of 41 +/- 9% bound for ZW800 carboxylate.

With the exception of ICG and SIDAG, which do not have functional handles for conjugation, the other dyes contain a carboxylate group that will not be present once the dye is conjugated to the targeting ligand. The optical properties of ICG are highly dependent on the microenvironment of the dye, which is impacted by plasma protein binding and partitioning[6,7]. Additionally, ICG is often delivered at very high doses, which can potentially saturate some binding sites in plasma and increase the free fraction. The estimates here are based on a subsaturating concentration of ICG[8].

For cellular uptake experiments, HEK-293 cells were used initially. However, the high blocking dose of integrin binder caused dissociation of these cells, so the cancer line MDA-MB-231 was used instead. Pinocytosis rates for macrophages and some tumor cell lines can approach 1.1×10^{-5}/s from fluid phase uptake[9]. Therefore, the measured uptake rates are postulated to be higher due to non-specific association with the cell surface and internalization.

3. Modeling Simplification and Analysis

Tissue concentration:

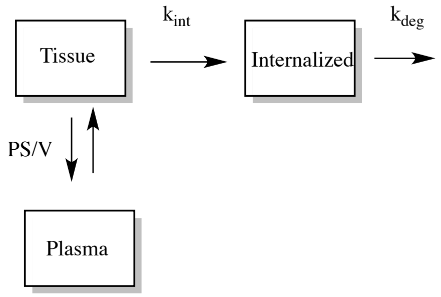

Assumptions:
-Equilibrium
-Well mixed
-"Tissue" compartment consists of bound and free imaging agent. In actuality, imaging agent leaves plasma and into the target tissue; once in the tissue, the free imaging agent binds to target receptors. We assume binding equilibrium is very fast compared to diffusion of the imaging agent out of the plasma. The model assumes the receptor in excess. For these two reasons: 1) fast equilibrium and 2) receptor in excess, target tissue and receptor-expressing cells are combined into one compartment.

Governing equations:
Assuming biexponential decay in plasma:

$$\frac{P_{plasma}}{P_{plasma,0}} = A\exp(-k_{\alpha}t) + B\exp(-k_{\beta}t)$$

In the tissue, the peptide can be either bound or free

$$R + P_{free} \rightleftharpoons P_{bound}$$

The binding affinity, K_d, can be used to relate bound, free, and receptor concentrations:

$$K_d = \frac{P_{free}R}{P_{bound}}$$

Assuming P_{tissue} is the total imaging agent concentration in the islet is the sum of P_{free} and P_{bound}. Assume receptor R is in excess, following equilibria equations are obtained.

$$P_{free} = \left(\frac{K_d}{R/\varepsilon + K_d}\right)P_{tissue}$$

$$P_{bound} = \left(\frac{R/\varepsilon}{R/\varepsilon + K_d}\right)P_{tissue}$$

The overall balance on extracellular islet concentration P_{islet} can be written

$$\frac{dP_{tissue}}{dt} = \frac{2PR_{cap}}{R_{Krogh}^2}\left((1-x)P_{plasma} - \frac{P_{free}}{\varepsilon}\right) - k_{\text{int}}P_{bound}$$

The initial condition is $P(t=0)_{tissue}=0$. Substitute $P_{plasma}(t)$, and the equation can be written in the form

$$\frac{dx}{dt}+ax=be^{-k_\alpha t}+ce^{-k_\beta t}$$

Multiplying both sides by the integrating factor e^{at} and reverse product rule the RHS to give

$$\frac{d}{dt}(xe^{at})=e^{at}(be^{-k_\alpha t}+ce^{-k_\beta t})$$

integration and application of the boundary condition gives (with appropriate coefficients)

$$P_{tissue}=(1-x)P_{plasma,0}\frac{2PR_{cap}}{R_{Krogh}^2}[\frac{A}{\Omega-k_\alpha}(e^{-k_\alpha t}-e^{-\Omega t})+\frac{B}{\Omega-k_\beta}(e^{-k_\beta t}-e^{-\Omega t})]$$

with

$$\Omega=\frac{2PR_{cap}}{\varepsilon R_{Krogh}^2}(\frac{K_d}{R/\varepsilon+K_d})+k_{\text{int}}(\frac{R/\varepsilon}{R/\varepsilon+K_d})$$

This will model the extracellular concentration of probe in the tissue.
To find the intracellular concentration, we use the governing equation for the intracellular compartment

$$\frac{dP_{\text{int}}}{dt}=k_{\text{int}}P_{bound}-k_{\text{deg}}P_{\text{int}}$$

Boundary condition is at t=0, $P_{int}=0$. With appropriate substitutions, the resulting ODE can be written in the form

$$\frac{dP_{\text{int}}}{dt}+k_{\text{deg}}P_{\text{int}}=be^{-k_\alpha t}+ce^{-k_\beta t}-de^{-\Omega t}$$

Multiplying both sides by the integrating factor $e^{k_{\text{deg}}t}$ and reverse product rule the RHS to give

$$\frac{d}{dt}(P_{\text{int}}e^{k_{\text{deg}}t})=e^{k_{\text{deg}}t}(be^{-k_\alpha t}+ce^{-k_\beta t}-de^{-\Omega t})$$

integration and application of the boundary condition gives (with appropriate coefficients)

$$P_{\text{int}}=\Phi\left[\frac{A}{(\Omega-k_\alpha)}\left(\frac{e^{-k_\alpha t}-e^{-k_{\text{deg}}t}}{k_{\text{deg}}-k_\alpha}+\frac{e^{-k_{\text{deg}}t}-e^{-\Omega t}}{k_{\text{deg}}-\Omega}\right)+\frac{B}{(\Omega-k_\beta)}\left(\frac{e^{-k_\beta t}-e^{-k_{\text{deg}}t}}{k_{\text{deg}}-k_\beta}+\frac{e^{-k_{\text{deg}}t}-e^{-\Omega t}}{k_{\text{deg}}-\Omega}\right)\right]$$

with

$$\Omega=\frac{2PR_{cap}}{\varepsilon R_{Krogh}^2}(\frac{K_d}{R/\varepsilon+K_d})+k_{\text{int}}(\frac{R/\varepsilon}{R/\varepsilon+K_d})$$

and

$$\Phi=k_{\text{int}}(1-x)P_{plasma,0}(\frac{R/\varepsilon}{R/\varepsilon+K_d})(\frac{2PR_{cap}}{R_{Krogh}^2})$$

Assumptions

Model assumptions	
1. Subsaturating dose	higher doses will lower TBR, so optimal assumption
2. No blood flow limitations	Important for tumors, which have slower blood flow rates. An extremely rapid clearance may not allow the tumor vessels to fill with probe.
3. Non-specific uptake is linear with concentration	There could be non-linear background uptake (e.g. target specific), but pinocytosis, non-specific binding, etc. are often linear.
Simplifying assumptions	
4. High affinity	Assumes all probe that enters tissue binds and does not dissociate, so optimal assumption. Internalization may help put a limit on the affinity, since once $k_e >> k_{off}$, all of the bound probe internalizes.
5. $k_{deg} << k_\alpha$ and $k_{deg} << k_{int}$	This is an optimal estimate to maximize the signal.
6. The imaging time is greater than k_α so the majority of probe is cleared from the blood	This is a optimal estimate. The signal in blood (both target and non-target) will lower the specific TBR since it is non-specific signal.
The combination of assumptions 4 and 5 above allows us to assume $k_{deg} \sim 0$ so $\exp(-k_{deg}t) \sim 1$ and $\exp(-k_\alpha t) \sim 0$	

Target Tissue

For the target tissue, starting with the model solution above:

At long times, all the blood and surface signal is gone, so only P_{int} determines the signal.

$$P_{\mathrm{int}} = \Phi\left[\frac{A}{(\Omega-k_\alpha)}\left(\frac{e^{-k_\alpha t}-e^{-k_{\deg}t}}{k_{\deg}-k_\alpha}+\frac{e^{-k_{\deg}t}-e^{-\Omega t}}{k_{\deg}-\Omega}\right)+\frac{B}{(\Omega-k_\beta)}\left(\frac{e^{-k_\beta t}-e^{-k_{\deg}t}}{k_{\deg}-k_\beta}+\frac{e^{-k_{\deg}t}-e^{-\Omega t}}{k_{\deg}-\Omega}\right)\right]$$

Assuming high affinity ($K_d \rightarrow 0$),

$$\Omega = k_{\mathrm{int}}$$

and

$$\Phi = k_{\mathrm{int}}(1-x)P_{plasma,0}\left(\frac{2PR_{cap}}{R_{Krogh}^2}\right)$$

$$P_{\mathrm{int}} = \Phi\left[\frac{A}{(k_{\mathrm{int}}-k_\alpha)}\left(\frac{e^{-k_\alpha t}-e^{-k_{\deg}t}}{k_{\deg}-k_\alpha}+\frac{e^{-k_{\deg}t}-e^{-k_{\mathrm{int}}t}}{k_{\deg}-k_{\mathrm{int}}}\right)+\frac{B}{(k_{\mathrm{int}}-k_\beta)}\left(\frac{e^{-k_\beta t}-e^{-k_{\deg}t}}{k_{\deg}-k_\beta}+\frac{e^{-k_{\deg}t}-e^{-k_{\mathrm{int}}t}}{k_{\deg}-k_{\mathrm{int}}}\right)\right]$$

If the imaging time is after clearance, and degradation is negligible

$$P_{\text{int}} = \Phi\left[\frac{A}{(k_{\text{int}}-k_\alpha)}\left(\frac{-1}{k_{\text{deg}}-k_\alpha}+\frac{1}{k_{\text{deg}}-k_{\text{int}}}\right)+\frac{B}{(k_{\text{int}}-k_\beta)}\left(\frac{-1}{k_{\text{deg}}-k_\beta}+\frac{1}{k_{\text{deg}}-k_{\text{int}}}\right)\right]$$

$$P_{\text{int}} = \Phi\left[\left(\frac{A}{(k_{\text{deg}}-k_\alpha)(k_{\text{deg}}-k_{\text{int}})}\right)+\left(\frac{B}{(k_{\text{deg}}-k_\beta)(k_{\text{deg}}-k_{\text{int}})}\right)\right]$$

Using assumption that $k_{\text{deg}} << k_{\text{alpha/beta}}$ and k_{int} and adding phi:

$$P_{\text{int}} = k_{\text{int}}(1-x)P_{plasma,0}\left(\frac{2PR_{cap}}{R_{Krogh}^2}\right)\left[\left(\frac{A}{(k_\alpha)(k_{\text{int}})}\right)+\left(\frac{B}{(k_\beta)(k_{\text{int}})}\right)\right]$$

$$P_{\text{int}} = (1-x)P_{plasma,0}\left(\frac{2PR_{cap}}{R_{Krogh}^2}\right)\left[\left(\frac{A}{k_\alpha}\right)+\left(\frac{B}{k_\beta}\right)\right]$$

$$P_{\text{int}} = (1-x)P_{plasma,0}\left(\frac{PS}{V}\right)\left[\left(\frac{A}{k_\alpha}\right)+\left(\frac{B}{k_\beta}\right)\right]$$

Non-target tissue

For non-target tissue, assuming first order internalization of probe non-specifically in the tissue. This in place of the high affinity assumption, but all other assumptions apply. Looking at long times after the probe has cleared from the blood:

$$P_{\text{int}} = \Phi\left[\frac{A}{(\Omega-k_\alpha)}\left(\frac{e^{-k_\alpha t}-e^{-k_{\text{deg}}t}}{k_{\text{deg}}-k_\alpha}+\frac{e^{-k_{\text{deg}}t}-e^{-\Omega t}}{k_{\text{deg}}-\Omega}\right)+\frac{B}{(\Omega-k_\beta)}\left(\frac{e^{-k_\beta t}-e^{-k_{\text{deg}}t}}{k_{\text{deg}}-k_\beta}+\frac{e^{-k_{\text{deg}}t}-e^{-\Omega t}}{k_{\text{deg}}-\Omega}\right)\right]$$

If the uptake is non-specific and linear with concentration, this is the same as a very low affinity antibody where the bound portion is linear with concentration (Kd → large):

$$\Omega = \frac{2PR_{cap}}{\varepsilon R_{Krogh}^2}\left(\frac{K_d}{R/\varepsilon + K_d}\right) + k_{\text{int}}\left(\frac{R/\varepsilon}{R/\varepsilon + K_d}\right) = \frac{2PR_{cap}}{\varepsilon R_{Krogh}^2} + k_{\text{int}}\left(\frac{R/\varepsilon}{K_d}\right)$$

The first term incorporates washout from the tissue for the unbound probe, and the second term is linear in concentration. The form is first order, but the mechanism is completely different, so we will substitute a new constant, $k_{\text{int,ns}}$, which stands for k internalization, non-specific (ns):

$$\Omega = \frac{2PR_{cap}}{\varepsilon R_{Krogh}^2} + k_{\text{int},ns}$$

We will also assume that the imaging time as after plasma clearance but before significant degradation (assumptions 5 and 6)

$$P_{\text{int}} = \Phi\left[\frac{A}{(\Omega - k_\alpha)}\left(\frac{-1}{k_{\deg} - k_\alpha} + \frac{1}{k_{\deg} - \Omega}\right) + \frac{B}{(\Omega - k_\beta)}\left(\frac{-1}{k_{\deg} - k_\beta} + \frac{1}{k_{\deg} - \Omega}\right)\right]$$

$$P_{\text{int}} = \Phi\left[\frac{A}{(\Omega - k_\alpha)}\left(\frac{\Omega - k_\alpha}{\left(k_{\deg} - k_\alpha\right)\left(k_{\deg} - \Omega\right)}\right) + \frac{B}{(\Omega - k_\beta)}\left(\frac{\Omega - k_\beta}{\left(k_{\deg} - k_\beta\right)\left(k_{\deg} - \Omega\right)}\right)\right]$$

$$P_{\text{int}} = \Phi\left[\left(\frac{A}{\left(k_{\deg} - k_\alpha\right)\left(k_{\deg} - \Omega\right)}\right) + \left(\frac{B}{\left(k_{\deg} - k_\beta\right)\left(k_{\deg} - \Omega\right)}\right)\right]$$

Substituting

$$k_{\text{int},ns} = k_{\text{int}}\left(\frac{R/\varepsilon}{K_d}\right)$$

similar to above:

$$\Phi = k_{\text{int},ns}(1-x)P_{plasma,0}\left(\frac{2PR_{cap}}{R_{Krogh}^2}\right)$$

Substituting

$$P_{\text{int}} = k_{\text{int},ns}(1-x)P_{plasma,0}\left(\frac{2PR_{cap}}{R_{Krogh}^2}\right)\left[\left(\frac{A}{\left(k_{\deg} - k_\alpha\right)\left(k_{\deg} - \Omega\right)}\right) + \left(\frac{B}{\left(k_{\deg} - k_\beta\right)\left(k_{\deg} - \Omega\right)}\right)\right]$$

Using assumption 5 (specifically that $k_{\deg} << k_{\text{int,ns}}$ and clearance, in this case

$$P_{\text{int}} = k_{\text{int},ns}(1-x)P_{plasma,0}\left(\frac{2PR_{cap}}{R_{Krogh}^2}\right)\left(\frac{1}{\left(k_\alpha\right)(\Omega)}\right)$$

$$P_{\text{int}} = k_{\text{int},ns}(1-x)P_{plasma,0}\left(\frac{2PR_{cap}}{R_{Krogh}^2}\right)\left[\left(\frac{A}{\left(k_\alpha\right)(\Omega)}\right) + \left(\frac{B}{\left(k_\beta\right)(\Omega)}\right)\right]$$

$$P_{\text{int}} = k_{\text{int},ns}(1-x)P_{plasma,0}\left(\frac{2PR_{cap}}{R_{Krogh}^2}\right)\frac{1}{\Omega}\left[\left(\frac{A}{\left(k_\alpha\right)}\right) + \left(\frac{B}{\left(k_\beta\right)}\right)\right]$$

$$P_{\text{int}} = (1-x)P_{plasma,0}\left(\frac{PS}{V}\right)\left[\left(\frac{A}{\left(k_\alpha\right)}\right) + \left(\frac{B}{\left(k_\beta\right)}\right)\right]\left(\frac{k_{\text{int},ns}}{\left(\frac{2PR_{cap}}{\varepsilon R_{Krogh}^2} + k_{\text{int},ns}\right)}\right)$$

Maximizing TBR:

$$TBR = \frac{\left(P_{\text{int}}\right)_{target}}{\left(P_{\text{int}}\right)_{non-target}} = \frac{(1-x)P_{plasma,0}\left(\frac{PS}{V}\right)\left[\left(\frac{A}{k_\alpha}\right)+\left(\frac{B}{k_\beta}\right)\right]}{(1-x)P_{plasma,0}\left(\frac{PS}{V}\right)\left[\left(\frac{A}{k_\alpha}\right)+\left(\frac{B}{k_\beta}\right)\right]\left(\frac{k_{\text{int},ns}}{\left(\frac{2PR_{cap}}{\varepsilon R_{Krogh}^2}+k_{\text{int},ns}\right)}\right)}$$

$$TBR = \left[\frac{\left(\frac{PS}{V}\right)_{target}}{\left(\frac{PS}{V}\right)_{non-target}}\right]\left[\frac{\frac{2PR_{cap}}{\varepsilon R_{Krogh}^2}+k_{\text{int},ns}}{k_{\text{int},ns}}\right]$$

The initial plasma concentration (dose) and plasma clearance cancel from the analysis. The PS/V terms do not cancel, since these are the permeability surface area products for the target and non-target tissues, which can be different.

The permeability surface area product on the right is for the non-target tissue:

$$TBR = \left[\frac{\left(\frac{PS}{V}\right)_{target}}{\left(\frac{PS}{V}\right)_{non-target}}\right]\left[\frac{\left(\frac{PS}{V}\right)_{non-target}+\varepsilon k_{\text{int},ns}}{\varepsilon k_{\text{int},ns}}\right]$$

If the washout rate is higher than non-specific internalization (which is critical for imaging), the ratio is:

$$TBR = \left[\frac{\left(\frac{PS}{V}\right)_{\text{target}}}{\varepsilon k_{\text{int},ns}}\right]$$

Since the physiology of the target tissue is difficult to manipulate, the probe should be designed to have minimal non-specific uptake.

Other notes:

a. If there is no non-specific uptake in the background tissue, then the background always goes to zero at long times. This assumption is likely not true and ignores the background from autofluorescence or instrument noise (for radioactivity).

b. Faster clearance helps to keep assumption 5 and 6 true (imaging after everything cleared from the blood but little degradation), which is not the case for antibodies, for example.

c. The internalization rate, k_{int}, can help if there is low expression of target in the tissue. The internalization and recycle will prevent the dose from reaching saturation (since super-saturating doses lower the TBR). In other words, it helps maintain assumption 1.

d. Lower affinity would increase washout from the target tissue, lowering the TBR.

e. The MW of the probe affects clearance, but this appears to cancel out for TBR. However, the permeability of the target and non-target organs are a function of MW. For certain tissues (such as tumors), maximizing the ratio of permeabilities between target and non-target tissue would maximize the TBR.

4. Capillary permeability data

Compound	MW (Da)	Radius (nm)	Organ	Permeability (*10^7 cm/s)	Vasculature	Reference
Dextran	3300	1.5	LS174T tumor	154	Fenestrated, immature	[10]
Mouse Fc fragment	25000	2.66	LS174T tumor	3.74	Fenestrated, immature	[11]
Mouse Fab fragment	25000	2.66	LS174T tumor	4.61	Fenestrated, immature	[11]
Dextran	10000	2.7	LS174T tumor	32	Fenestrated, immature	[10]
Ovalbumin	45000	3.24	LS174T tumor	5.77	Fenestrated, immature	[11]
Evans blue albumin	66000	3.55	A-07 Tumor	15	Fenestrated, immature	[12]
Evans blue albumin	66000	3.55	R-18 Tumor	11	Fenestrated, immature	[12]
Evans blue albumin	66000	3.55	U-25 Tumor	9	Fenestrated, immature	[12]
Concanavalin A	104000	4.28	LS174T tumor	1.53	Fenestrated, immature	[11]
Mouse F(ab')2 fragment	110000	4.36	LS174T tumor	1.51	Fenestrated, immature	[11]
Mouse igG	160000	4.94	LS174T tumor	2.82	Fenestrated, immature	[11]
Dextran	40000	5	LS174T tumor	9.5	Fenestrated, immature	[10]
Dextran	70000	6.5	LS174T tumor	9.8	Fenestrated, immature	[10]
Dextran	2000000	22.1	LS174T tumor	1.7	Fenestrated, immature	[10]
Liposome	-	45	LS174T tumor	0.2	Fenestrated, immature	[11]
Liposome	-	60	LS174T tumor	0.155	Fenestrated, immature	[13]
Na	23	0.1	Kidney	7600	Fenestrated	[14]
Na	23	0.1	Pancreas	3600	Fenestrated	[15]
Urea	60	0.18	Kidney	36000	Fenestrated	[14]
Cr-EDTA	357	0.22	Pancreas	2200	Fenestrated	[15]
B12	1353	0.85	Pancreas	1100	Fenestrated	[15]
Inulin	5000	1.52	Kidney	1440	Fenestrated	[16]
Insulin	6000	1.66	Pancreas	348	Fenestrated	[15]
Albumin	66000	3.55	Kidney	71	Fenestrated	[17]
Albumin	66000	3.55	Pancreas	26	Fenestrated	[15]
Hydrophilic solute	-	0.24	Muscle	350	Non-fenestrated	[18]
Hydrophilic solute	-	0.26	Muscle	270	Non-fenestrated	[18]
Hydrophilic solute	-	0.44	Muscle	69	Non-fenestrated	[18]
Hydrophilic solute	-	0.58	Muscle	51	Non-fenestrated	[18]
Hydrophilic	-	1.5	Muscle	11	Non-	[18]

solute					fenestrated	
Hydrophilic solute	-	3.6	Muscle	0.47	Non-fenestrated	[18]
Hexose	180	0.42	Human forearm	130	Non-fenestrated	[19]
Hexose	180	0.42	Dog heart	93	Non-fenestrated	[19]
Sucrose	342	0.52	Human forearm	87	Non-fenestrated	[19]
Sucrose	342	0.52	Dog heart	71.5	Non-fenestrated	[19]
Sucrose EDTA	342	0.52	Cardiac muscle	350	Non-fenestrated	[20]
Sucrose EDTA	342	0.52	Skeletal muscle	140	Non-fenestrated	[21]
Raffinose	504	0.56	Human forearm	54.6	Non-fenestrated	[19]
Inulin	5300	1.52	Human forearm	9.3	Non-fenestrated	[19]
Inulin	5300	1.52	Dog heart	24	Non-fenestrated	[19]
Inulin	5300	1.52	Cardiac muscle	40	Non-fenestrated	[20]
Inulin	5500	1.52	Skeletal muscle	20	Non-fenestrated	[20]
α-Globulin	45000	3.55	Dog heart	0.606	Non-fenestrated	[19]
Albumin	66000	3.55	Dog paw	0.519	Non-fenestrated	[19]
Albumin	66000	3.55	Dog heart	0.313	Non-fenestrated	[19]
Albumin	66000	3.55	Dog intestine	0.304	Non-fenestrated	[19]

Table S1. Capillary permeability data for various compounds in different types of tissue. All permeability (P) values are experimentally determined and reported in literature as effective permeability, which account for convective and diffusive contributions. Molecular radii of compounds for kidney and pancreas permeability were compiled from literature[22,23]. For tumor permeability, molecular radii of compounds were calculated based on previously reported methods for globular proteins and PEG chains when not stated in the reference[24]. Vasculature type, whether fenestrated or non-fenestrated, followed criteria listed by Sarin et al[25].

Organ flow rate data

Organ	Plasma flow (mL/min)	Organ volume (mL)	Organ mass %	References
Bone	0.17	1.5	10.73	[26,27]
Heart	0.28	0.28	0.5	[27,28]
Kidney	0.8	0.298	1.67	[26,27]
Liver	1.1	0.951	5.49	[27,28]
Lung	4.38	0.191	0.73	[26,27]
Muscle	0.8	7.9	38.4	[27,28]
Spleen	0.05	0.1	0.50	[26]

Table S2. Plasma flow rate, organ volume, and organ mass % were compiled from the literature. To convert plasma flow rates to mL/min/g, organ masses were used. Organ masses were either reported or calculated using organ mass % and total animal mass of 25 g for mouse data[27]. For tumor, a literature reported value of 0.1 mL/min/g was used as the blood flow rate and converted to plasma flow rate assuming a hematocrit of 0.45[29]. Endocrine and exocrine pancreas plasma flow rates were reported in literature[30].

5. Simulations values

Islet simulation parameters data

Symbol	Parameter	Value	Notes	Reference
C_0	Initial Plasma Conc	1 fM	Dose dependent	
Q	Blood flow	0.029mL/s/g	Not rate limiting	[15,31]
v	Vessel velocity	0.6 mm/s		[32]
[T]	GLP-1 Receptor Conc	Varied		
P	Permeability	$2.1x10^{-5}$ cm/s	10-fold higher than non-fenestrated normal tissue	[15,33]
k_{on}	Binding rate	$1.4x10^{6}$ /M/s		[34]
D	Diffusion Coefficient	100 um^2/s	Not rate-limiting	[24]
S/V	Capillary surface area to tissue volume	505 cm^2/cm^3	Extremely high in islets	[35]
ε	Void fraction	0.1	Interstitial space in pancreas (adapted from insulin)	[15]
R_{cap}	Capillary radius	3 μm		[36]
k_{deg}	Residualization rate	0.012 /hr	Fluorescent dye specific	[37]
A	Fraction fast	0.91		[38]
k_{fast}	Alpha clearance	0.174 min^{-1}		[38]
k_{slow}	Beta clearance	0.0169 min^{-1}		[38]
V_{plasma}	Plasma volume	1.4 mL		[39]
Kd	Dissociation constant	Varied		
k_e	Receptor internalization	Varied		
ρ	Tissue density	1000 g/L		
$Plasma_{Bound}$	Fraction Plasma protein bound	0.43	Measured	
Simulation time		3 h		

Table S3. Parameters used for determining endocrine pancreas uptake of various GLP-1R ligands.

Tumor simulation parameters data

Symbol	Parameter	Value	Notes	References
C_0	Initial Plasma Conc	1 fM	Dose dependent	
Q	Blood flow	0.0016 mL/s/g	Not rate limiting	[40]
[T]	Receptor Conc	Varied		
P	Permeability	$8x10^{-6}$ cm/s	Fig. 2	Fig. 2
k_{on}	Binding rate	$4.4x10^{6}$ /M/s		[41]
D	Diffusion Coefficient	100 um^2/s	Not rate-limiting	[24]
R_{krogh}	Krogh cylinder radius	60 um		[42]
ε	Void fraction	0.2		[43]
R_{cap}	Capillary radius	10 μm		[44]
k_{deg}	Residualization rate	$4x10^{-6}$ s^{-1}	In^{111} half life of 48h	[45]
A	Fraction fast	0.9972		[41,46]
k_{fast}	Alpha clearance	2.83 h^{-1}		[41,46]
k_{slow}	Beta clearance	0.03 h^{-1}		[41,46]
V_{plasma}	Plasma volume	1.4 mL		[39]
K_d	Dissociation constant	Varied		
k_e	Internalization	Varied		
ρ	Tissue density	1000 g/L		
$Plasma_{Bound}$		0.43	Assumed similar PPB as exendin	
Simulation time		4 h		

Table S4. Parameters used for determining tumor uptake for various affibody molecules.

HER2 and GLP-1R ligand properties data

Compound	K_d (M)	k_e (s^{-1})	[T] (M)	%ID/g (measured)	Radiolabel	Receptor saturation	Reference
ZHER2:2395	$2.7*10^{-11}$	$3.9*10^{-6}$	$1*10^{-6}$	15	^{99m}Tc	N	[47-49]
ZHER2:4	$5.0*10^{-8}$	$3.9*10^{-6}$	$1*10^{-6}$	2.4	^{125}I	N	[46,48,49]
ZHER2:342	$2.2*10^{-11}$	$3.9*10^{-6}$	$1*10^{-6}$	9.46	^{125}I	N	[46,48,49]
A1-ZHER2:S1	$1.3*10^{-10}$	$3.9*10^{-6}$	$1*10^{-6}$	16	^{111}In	N	[48-50]
K50-ZHER2:S1	$1.1*10^{-10}$	$3.9*10^{-6}$	$1*10^{-6}$	13	^{111}In	N	[48-50]
K58-ZHER2:S1	$9.7*10^{-11}$	$3.9*10^{-6}$	$1*10^{-6}$	15	^{111}In	N	[48-50]
PEP05541	$1.2*10^{-10}$	$3.9*10^{-6}$	$1*10^{-6}$	12.9	^{111}In	N	[48,49,51]
PEP05838	$1.6*10^{-10}$	$3.9*10^{-6}$	$1*10^{-6}$	15.5	^{111}In	N	[48,49,51]
PEP07127	$3.8*10^{-9}$	$3.9*10^{-6}$	$1*10^{-6}$	14.3	^{111}In	N	[48,49,51]
ZHER2:2395	$2.7*10^{-11}$	$3.9*10^{-6}$	$2.5*10^{-8}$	6.9	^{99m}Tc	Y	[47,49,52]
ZHER2:342	$2.7*10^{-11}$	$3.9*10^{-6}$	$2.5*10^{-8}$	19	^{111}In	N	[49,52,53]
PEP05541	$1.2*10^{-10}$	$3.9*10^{-6}$	$2.5*10^{-8}$	7.4	^{111}In	N	[49,51,52]
PEP05838	$1.6*10^{-10}$	$3.9*10^{-6}$	$2.5*10^{-8}$	10.8	^{111}In	N	[49,51,52]
PEP07127	$3.8*10^{-9}$	$3.9*10^{-6}$	$2.5*10^{-8}$	1.71	^{111}In	N	[49,51,52]
800CW monomer	$2.6*10^{-9}$	0.002	$6.3*10^{-8}$	50	-	N	[38]
800CW dimer	$8.3*10^{-9}$	0.002	$6.3*10^{-8}$	45.7	-	N	[38]
800CW trimer	$2.1*10^{-8}$	0.002	$6.3*10^{-8}$	24	-	N	[38]

Table S5. Previously published data on tumor and endocrine pancreas uptake. For tumor, several affibody probes were considered in addition to various radiolabels. For islet targeting, three exendin-based compounds were considered. For predicted uptake values, dissociation constants were recalculated assuming constant activation energy to account for temperature differences between affinity measurements and the *in vivo* experiments.

6. Impact of vascular density on simulations

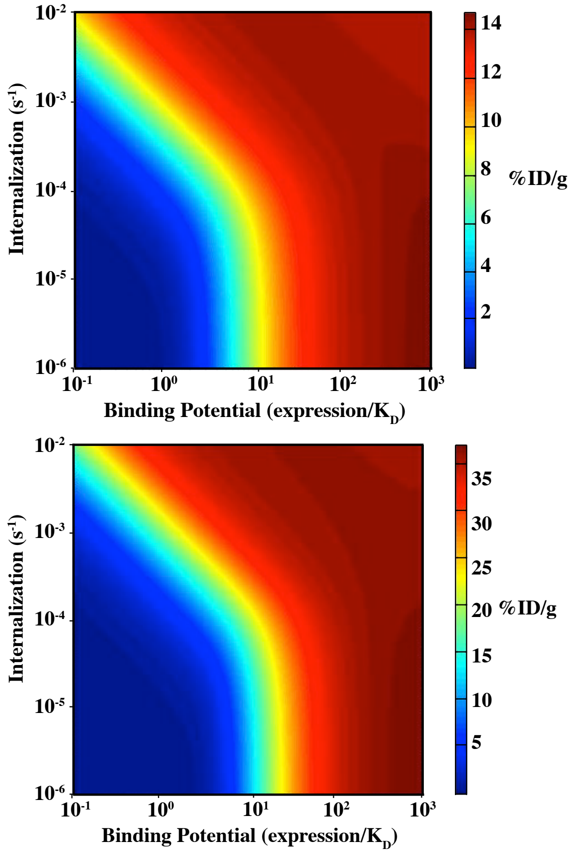

Figure S1. Contour plots for tumor uptake of a small peptide to demonstrate the effect of blood vessel surface area to volume (S/V). Top plot uses an S/V value of 60 cm^{-1}; bottom plot uses an S/V value of 200 cm^{-1}. Two noticeable differences between the plots include 1) a higher maximum tumor uptake with a greater S/V (top) and 2) near-maximum uptake occurs at a higher binding potential (higher expression or lower affinity) for greater S/V.

7. Probe synthesis and characterization

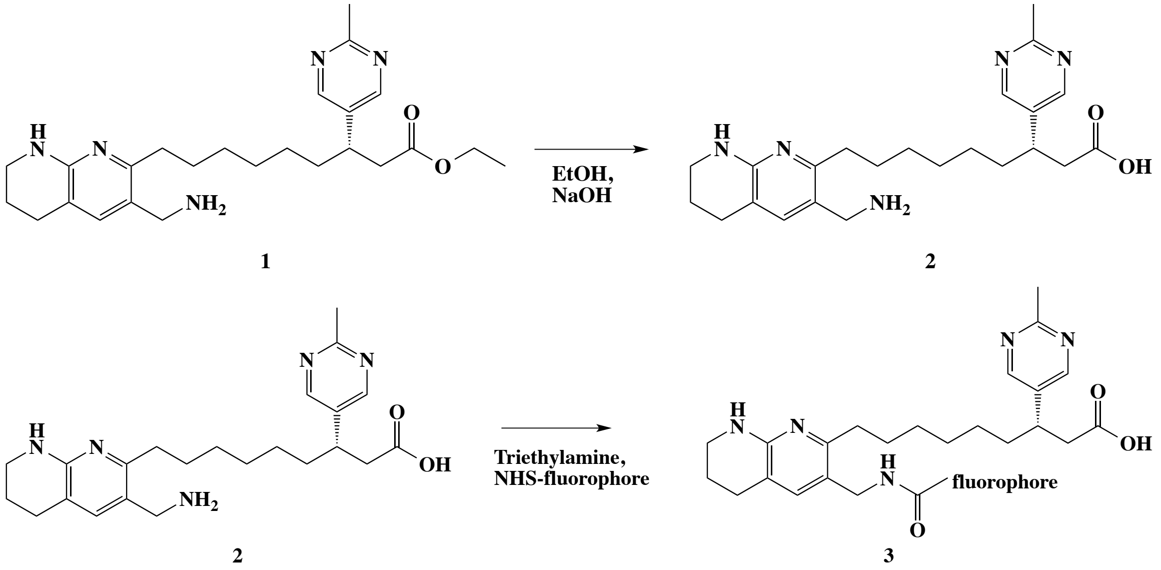

Figure S2. Reaction scheme for generating fluorescent αvβ3 targeting ligands. Hydrolysis (top) yields a carboxylic acid. Primary amine (bottom) reacts with NHS ester on various fluorophores. **1** was modified from Coleman et al.[54] and Kossodo et al.[55] and obtained from ChemPartner.

Purification and characterization of fluorescent conjugates

Compound	Mobile phase	HPLC method	Expected MW	Observed MW	Ionization	Purity
800CW conjugate	A: 50 mM TEAA in H_2O<br>B: MeCN	25% B 0-6 min,<br>25-45% B 6-24 min	1394.70	1395.922	MALDI-TOF	95.0%
AF680 conjugate	A: 0.1 % TFA in H_2O<br>B: 0.1 % TFA in MeCN	5-30% B 0-12 min,<br>30-60% B 12-16 min	1250.35	1253.314	MALDI-TOF	96.5%
B650 conjugate	A: 0.1 % TFA in H_2O<br>B: 0.1 % TFA in MeCN	25-95% B 0-15 min,<br>95% B 15-16 min	940.92	940.1	MALDI-TOF	87.5%
ZW800 conjugate	A: 0.1 % TFA in H_2O<br>B: 0.1 % TFA in MeCN	10-30% B 0-10 min,<br>30% B 10-14 min,<br>30-50% B 14-20 min	1337.77	1335.6817	ESI	90.2%

Table S6: Mobile phase and HPLC gradients are listed in the above table. Due to difficulty of ionization on ESI, certain conjugates were characterized using MALDI-TOF.

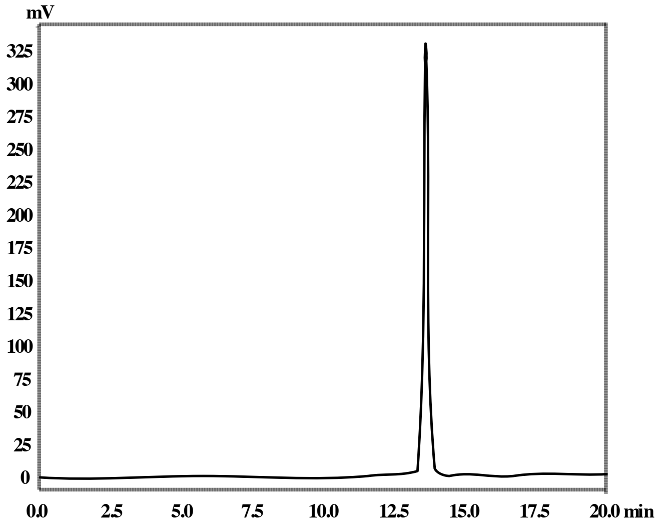

Figure S3. HPLC chromatogram (254 nm) for purified 800CW conjugate.

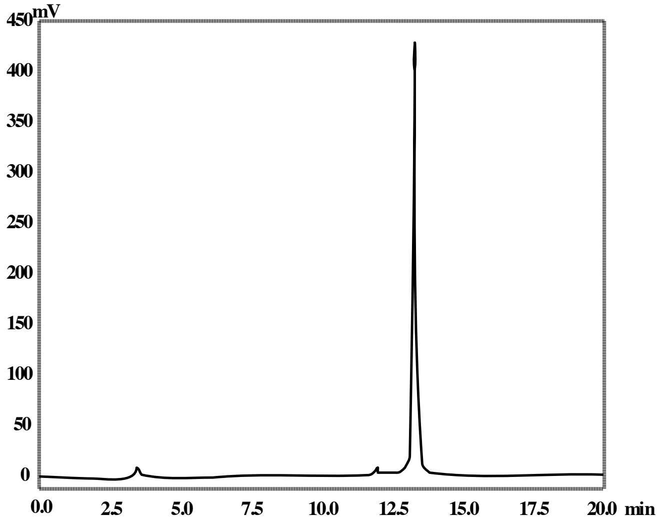

Figure S4. HPLC chromatogram (254 nm) for purified AF680 conjugate.

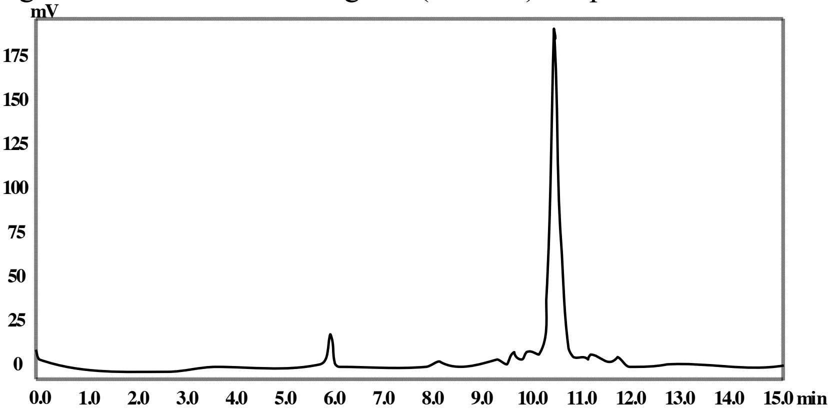

Figure S5. HPLC chromatogram (254 nm) for purified ZW800 conjugate.

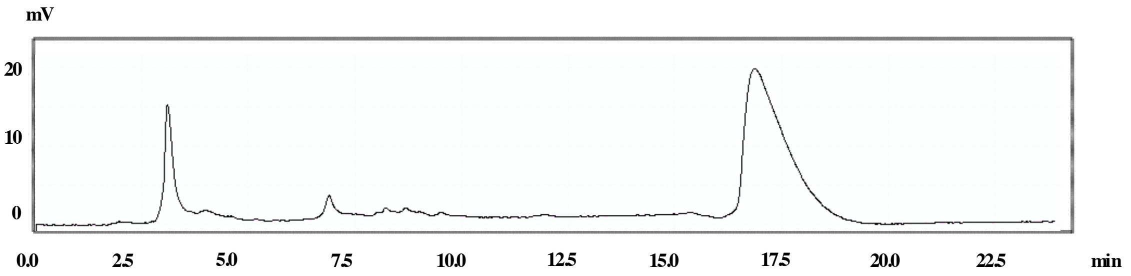

Figure S6. HPLC chromatogram (254 nm) for purified B650 conjugate. The peak at 3.5 min is DMSO used to dissolve the extremely lipophilic conjugate.

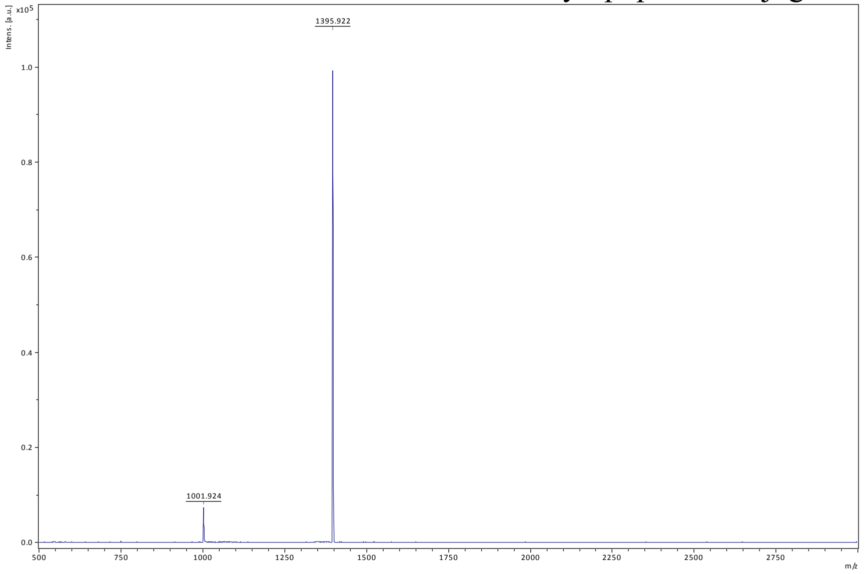

Figure S7. MALDI for the IRDye 800CW conjugate.

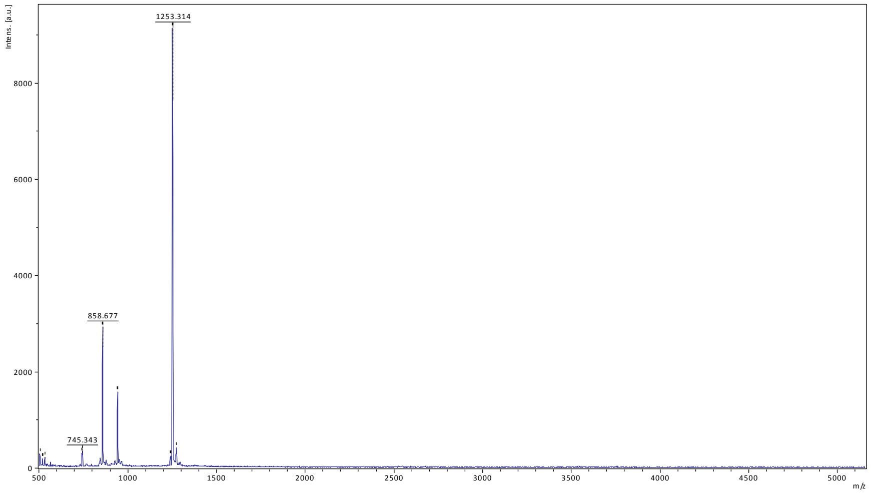

Figure S8. MALDI for the Alexa Fluor 680 conjugate.

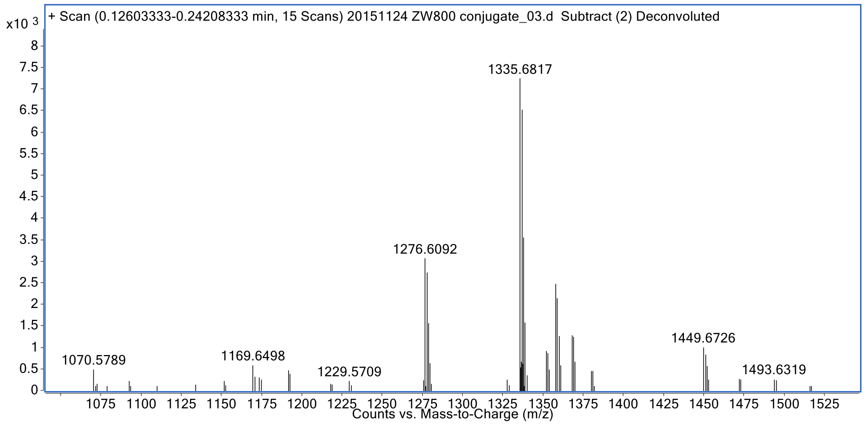

Figure S9. ESI for the ZW800 conjugate.

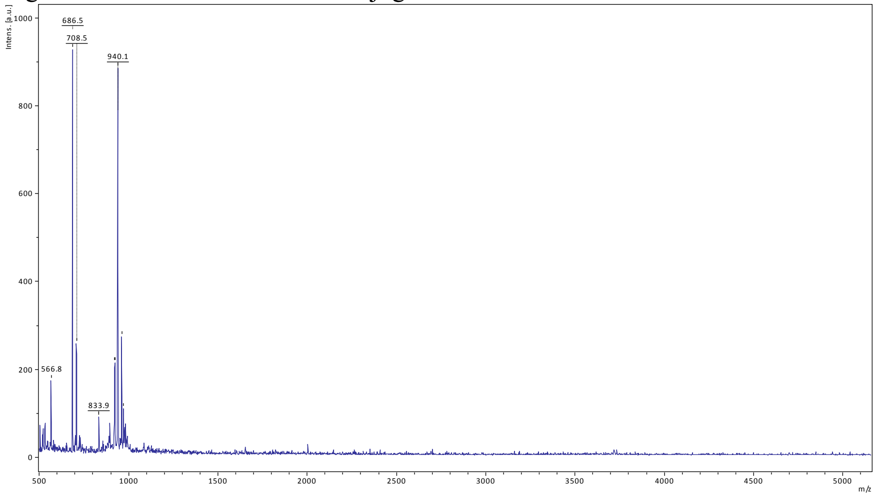

Figure S10. MALDI for the BODIPY 650 conjugate.

8. Charge versus Tumor to Muscle Ratio

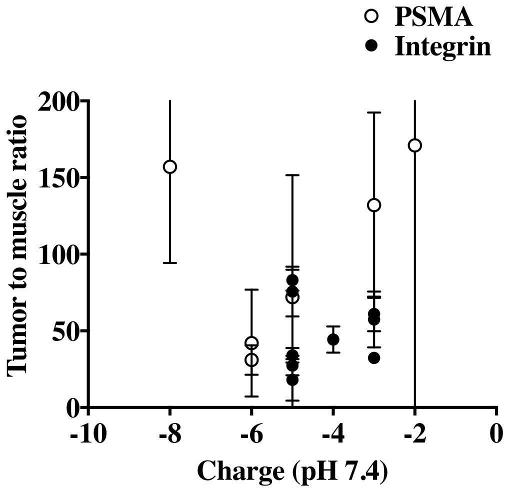

A correlation between charge and TBR was also investigated using uptake values from the PSMA and integrin targeting compounds. Figure (above) shows a plot of TBR versus the overall charge of all compounds at pH 7.4. The plot suggests no evident trend between charge and uptake (R^2=0.05, R^2=0.01 for PSMA and integrin binders, respectively) likely due to the importance of chelation chemistry in determining overall molecular charge and highlighting one challenge in prediction from pure structure.

9. Exendin non-specific uptake

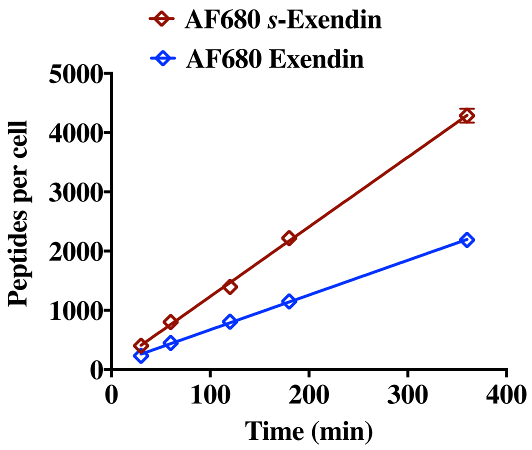

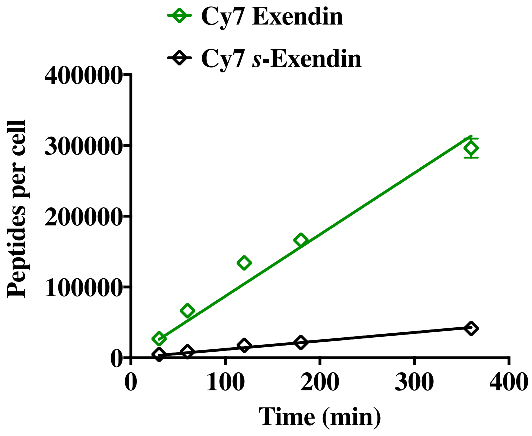

HT1080 cells were grown in 24-well tissue culture plates. After adhering overnight, cells were incubated with 100 nM of different fluorescent exendin conjugates at 37°C. Fluorescent exendin conjugates are conjugated with either lipophilic or hydrophilic fluorophores to increase or decrease non-specific plasma protein and membrane interactions. After the each time point, cells were harvested and analyzed on an Attune Focusing cytometer to quantify the intracellular signal as a function of time. A blocking control was performed with excess non-fluorescent exendin to demonstrate the fluorescent signal is from non-specific uptake. The number of internalized molecules per cell as a function of time was used to calculate the non-specific uptake rate.

Supplemental References

1 Harris, T. D. *et al.* Design, synthesis, and evaluation of radiolabeled integrin alpha v beta 3 receptor antagonists for tumor imaging and radiotherapy. *Cancer Biother Radiopharm* **18**, 627-641 (2003).

2 Onthank, D. *Prediction of "First Dose in Human" for Radiopharmaceuticals / Imaging Agents Based on Allometric Scaling of Pharmacokinetics in Pre-Clinical Animal Models* Doctor of Philosophy thesis, Worcester Polytechnic Institute, (2005).

3 Kaliss, N. & Pressman, D. Plasma and blood volumes of mouse organs as determined with radioactive iodoproteins. *Proceedings of the Society for Experimental Biology and Medicine* **75**, 16-20 (1950).

4 Konikowski, T., Hayhie, T. P., Glenn, H. J. & Farr, L. E. Iodohippurate sodium 131-I (OIH) clearance in mice bioassay of radiopharmaceuticals. *Proc Soc Exp Biol Med* **137**, 1343-1351 (1971).

5 Russell, C. D., Rowell, K. & Scott, J. W. Quality control of technetium-99m DTPA: correlation of analytic tests with in vivo protein binding in man. *J Nucl Med* **27**, 560-562 (1986).

6 Landsman, M. L., Kwant, G., Mook, G. A. & Zijlstra, W. G. Light-absorbing properties, stability, and spectral stabilization of indocyanine green. *J Appl Physiol* **40**, 575-583 (1976).

7 Mordon, S., Devoisselle, J. M., Soulie-Begu, S. & Desmettre, T. Indocyanine green: physicochemical factors affecting its fluorescence in vivo. *Microvasc Res* **55**, 146-152, doi:10.1006/mvre.1998.2068 (1998).

8 Ott, P. Hepatic elimination of indocyanine green with special reference to distribution kinetics and the influence of plasma protein binding. *Pharmacol Toxicol* **83 Suppl 2**, 1-48 (1998).

9 Thurber, G. M., Figueiredo, J. L. & Weissleder, R. Multicolor fluorescent intravital live microscopy (FILM) for surgical tumor resection in a mouse xenograft model. *PLoS ONE* **4**, e8053 (2009).

10 Dreher, M. R. *et al.* Tumor vascular permeability, accumulation, and penetration of macromolecular drug carriers. *J. Natl. Cancer Inst.* **98**, 335-344 (2006).

11 Yuan, F. *et al.* Vascular permeability in a human tumor xenograft: molecular size dependence and cutoff size. *Cancer Res* **55**, 3752-3756 (1995).

12 Graff, B. A., Bjornaes, I. & Rofstad, E. K. Macromolecule uptake in human melanoma xenografts. relationships to blood supply, vascular density, microvessel permeability and extracellular volume fraction. *Eur J Cancer* **36**, 1433-1440 (2000).

13 Yuan, F. *et al.* Microvascular permeability and interstitial penetration of sterically stabilized (stealth) liposomes in a human tumor xenograft. *Cancer Res* **54**, 3352-3356 (1994).

14 Pallone, T. L., Work, J., Myers, R. L. & Jamison, R. L. Transport of sodium and urea in outer medullary descending vasa recta. *J. Clin. Invest.* **93**, 212-222 (1994).

15 Sweiry, J. H. & Mann, G. E. Pancreatic Microvascular Permeability in Cerulein-Induced Acute-Pancreatitis. *Am J Physiol* **261**, G685-G692 (1991).
16 Crone, C. The Permeability of Capillaries in Various Organs as Determined by Use of the 'Indicator Diffusion' Method. *Acta Physiol Scand* **58**, 292-305 (1963).
17 Turner, M. R. & Pallone, T. L. Hydraulic and diffusional permeabilities of isolated outer medullary descending vasa recta from the rat. *Am J Physiol* **272**, H392-400 (1997).
18 Michel, C. C. & Curry, F. E. Microvascular permeability. *Physiol Rev* **79**, 703-761 (1999).
19 Dedrick, R. L. & Flessner, M. F. Pharmacokinetic considerations on monoclonal antibodies. *Prog Clin Biol Res* **288**, 429-438 (1989).
20 Crone, C., D.G. Levitt. Capillary permeability to small solutes. *Handbook of Physiology. The Cardiovascular System. Microcirculation.* (1984).
21 Paaske, W. P. Capillary permeability in skeletal muscle. *Acta Physiol Scand* **101**, 1-14 (1977).
22 Heyrovska, R. Effective Radii of Alkali-Halide Ions in Aqueous-Solutions, Crystals and in the Gas-Phase and the Interpretation of Stokes Radii. *Chem. Phys. Lett.* **163**, 207-211 (1989).
23 Schultz, S. G. & Solomon, A. K. Determination of the effective hydrodynamic radii of small molecules by viscometry. *J Gen Physiol* **44**, 1189-1199 (1961).
24 Schmidt, M. M. & Wittrup, K. D. A modeling analysis of the effects of molecular size and binding affinity on tumor targeting. *Mol Cancer Ther* **8**, 2861-2871 (2009).
25 Sarin, H. Physiologic upper limits of pore size of different blood capillary types and another perspective on the dual pore theory of microvascular permeability. *J Angiogenes Res* **2**, 14 (2010).
26 Gerlowski, L. E. & Jain, R. K. Physiologically based pharmacokinetic modeling: principles and applications. *J Pharm Sci* **72**, 1103-1127 (1983).
27 Brown, R. P., Delp, M. D., Lindstedt, S. L., Rhomberg, L. R. & Beliles, R. P. Physiological parameter values for physiologically based pharmacokinetic models. *Toxicol Ind Health* **13**, 407-484 (1997).
28 Barbee, R. W., Perry, B. D., Re, R. N. & Murgo, J. P. Microsphere and dilution techniques for the determination of blood flows and volumes in conscious mice. *Am J Physiol* **263**, R728-733 (1992).
29 Vaupel, P., Kallinowski, F. & Okunieff, P. Blood flow, oxygen and nutrient supply, and metabolic microenvironment of human tumors: a review. *Cancer Res* **49**, 6449-6465 (1989).
30 Carlsson, P. O., Berne, C. & Jansson, L. Angiotensin II and the endocrine pancreas: effects on islet blood flow and insulin secretion in rats. *Diabetologia* **41**, 127-133 (1998).
31 Friedman, H. S., Lowery, R., Shaughnessy, E. & Scorza, J. The Effects of Ethanol on Pancreatic Blood-Flow in Awake and Anesthetized Dogs. *Proc Soc Exp Biol Med* **174**, 377-382 (1983).

32 Menger, M. D., Vajkoczy, P., Leiderer, R., Jager, S. & Messmer, K. Influence of experimental hyperglycemia on microvascular blood perfusion of pancreatic islet isografts. *J. Clin. Invest.* **90**, 1361-1369 (1992).

33 Kvietys, P. R., Perry, M. A. & Granger, D. N. Permeability of Pancreatic Capillaries to Small Molecules. *Am J Physiol* **245**, G519-G524 (1983).

34 Widmann, C., Dolci, W. & Thorens, B. Agonist-induced internalization and recycling of the glucagon-like peptide-1 receptor in transfected fibroblasts and in insulinomas. *The Biochemical Journal* **310 (Pt 1)**, 203-214 (1995).

35 Keliher, E. J., Reiner, T., Thurber, G. M., Upadhyay, R. & Weissleder, R. Efficient18F-Labeling of Synthetic Exendin-4 Analogues for Imaging Beta Cells. *ChemistryOpen* **1**, 177-183 (2012).

36 Kirkpatrick, J. P., Brizel, D. M. & Dewhirst, M. W. A mathematical model of tumor oxygen and glucose mass transport and metabolism with complex reaction kinetics. *Radiat Res* **159**, 336-344 (2003).

37 Cilliers, C., Liao, J., Atangcho, L. & Thurber, G. M. Residualization Rates of Near-Infrared Dyes for the Rational Design of Molecular Imaging Agents. *Mol. Imaging Biol.* (2015).

38 Zhang, L. & Thurber, G. M. Quantitative Impact of Plasma Clearance and Down-regulation on GLP-1 Receptor Molecular Imaging. *Mol. Imaging Biol.* (2015).

39 Kaliss, N. & Pressman, D. Plasma and Blood Volumes of Mouse Organs, as Determined with Radioactive Iodoproteins. *Proc Soc Exp Biol Med* **75**, 16-20 (1950).

40 Baxter, L. T., Zhu, H., Mackensen, D. G. & Jain, R. K. Physiologically based pharmacokinetic model for specific and nonspecific monoclonal antibodies and fragments in normal tissues and human tumor xenografts in nude mice. *Cancer Res* **54**, 1517-1528 (1994).

41 Tolmachev, V. *et al.* 111In-benzyl-DTPA-ZHER2:342, an affibody-based conjugate for in vivo imaging of HER2 expression in malignant tumors. *J Nucl Med* **47**, 846-853 (2006).

42 Baish, J. W. *et al.* Role of tumor vascular architecture in nutrient and drug delivery: an invasion percolation-based network model. *Microvasc Res* **51**, 327-346 (1996).

43 Krol, A., Nagaraj, S., Dewhirst, M. & Yuan, F. Available volume fraction of macromolecules in tumor tissues. *FASEB J* **14**, A167-A167 (2000).

44 Thurber, G. M., Zajic, S. C. & Wittrup, K. D. Theoretic criteria for antibody penetration into solid tumors and micrometastases. *J Nucl Med* **48**, 995-999 (2007).

45 Stein, R., Goldenberg, D. M., Ong, G. L., Thorpe, S. R. & Mattes, M. J. Manipulation of blood clearance to optimize delivery of residualizing label-antibody conjugates to tumor cells in vivo. *J Nucl Med* **38**, 1392-1400 (1997).

46 Orlova, A. *et al.* Tumor Imaging using a picomolar affinity HER2 binding affibody molecule. *Cancer Res* **66**, 4339-4348 (2006).

47 Ahlgren, S. *et al.* Targeting of HER2-Expressing Tumors with a Site-Specifically Tc-99m-Labeled Recombinant Affibody Molecule, Z(HER2:2395), with C-Terminally Engineered Cysteine. *J Nucl Med* **50**, 781-789 (2009).

48 Engfeldt, T. *et al.* Imaging of HER2-expressing tumours using a synthetic Affibody molecule containing the Tc-99m-chelating mercaptoacetyl-glycyl-glycyl-glycyl (MAG3) sequence. *Eur. J. Nucl. Med. Mol. Imaging* **34**, 722-733 (2007).

49 Wallberg, H. & Orloval, A. Slow internalization of anti-HER2 synthetic Affibody monomer In-111-DOTA-Z(HER2 : 342-pep2): Implications for development of labeled tracers. *Cancer Biother. Radiopharm.* **23**, 435-442 (2008).

50 Honarvar, H. *et al.* Position for Site-Specific Attachment of a DOTA Chelator to Synthetic Affibody Molecules Has a Different Influence on the Targeting Properties of Ga-68-Compared to In-111-Labeled Conjugates. *Mol. Imaging* **13** (2014).

51 Tolmachev, V. *et al.* Tumor Targeting Using Affibody Molecules: Interplay of Affinity, Target Expression Level, and Binding Site Composition. *J Nucl Med* **53**, 953-960 (2012).

52 Milenic, D. E. *et al.* Targeting of HER2 antigen for the treatment of disseminated peritoneal disease. *Clin. Cancer Res.* **10**, 7834-7841 (2004).

53 Tolmachev, V. *et al.* Optimal specific radioactivity of anti-HER2 Affibody molecules enables discrimination between xenografts with high and low HER2 expression levels. *Eur. J. Nucl. Med. Mol. Imaging* **38**, 531-539 (2011).

54 Coleman, P. J. *et al.* Nonpeptide alpha(v)beta(3) antagonists. Part 11: Discovery and preclinical evaluation of potent alpha v beta(3) antagonists for the prevention and treatment of osteoporosis. *J Med Chem* **47**, 4829-4837 (2004).

55 Kossodo, S. *et al.* Dual In Vivo Quantification of Integrin-targeted and Protease-activated Agents in Cancer Using Fluorescence Molecular Tomography (FMT). *Mol. Imaging Biol.* **12**, 488-499 (2010).
